# *PsySuite*: An android application designed to perform multimodal psychophysical testing

**DOI:** 10.3758/s13428-024-02475-4

**Published:** 2024-08-13

**Authors:** Alberto Inuggi, Nicola Domenici, Alessia Tonelli, Monica Gori

**Affiliations:** U-VIP, Unit for Visually Impaired People, Istituto Italiano Di Tecnologia, Via Enrico Melen, 83, 16152 Genoa, GE Italy

**Keywords:** Android, Psychophysics, Double-flash illusion, Perception

## Abstract

In behavioral sciences, there is growing concern about the inflation of false-positive rates due to the amount of under-powered studies that have been shared in the past years. While problematic, having the possibility to recruit (lots of) participants (for a lot of time) is realistically not achievable for many research facilities. Factors that hinder the reaching of optimal sample sizes are, to name but a few, research costs, participants’ availability and commitment, and logistics. We challenge these issues by introducing *PsySuite*, an Android app designed to foster a remote approach to multimodal behavioral testing. To validate *PsySuite*, we first evaluated its ability to generate stimuli appropriate to rigorous psychophysical testing, measuring both the app’s accuracy (i.e., stimuli’s onset, offset, and multimodal simultaneity) and precision (i.e., the stability of a given pattern across trials), using two different smartphone models. We then evaluated *PsySuite*’s ability to replicate perceptual performances obtained using a classic psychophysical paradigm, comparing sample data collected with the app against those measured via a PC-based setup. Our results showed that *PsySuite* could accurately reproduce stimuli with a minimum duration of 7 ms, 17 ms, and 30 ms for the auditory, visual, and tactile modalities, respectively, and that perceptual performances obtained with *PsySuite* were consistent with the perceptual behavior observed using the classical setup. Combined with the high accessibility inherently supported by *PsySuite*, here we ought to share the app to further boost psychophysical research, aiming at setting it to a cheap, user-friendly, and portable level.

**Supplementary Information:**

The online version contains supplementary material available at 10.3758/s13428-024-02475-4.

## Introduction

The term 'psychophysics' was first introduced in the middle of the nineteenth century by the German scientist Gustav Theodor Fechner, who defined its principles by unraveling the already-heated debate about the distinctions between matter and mind (Fechner, 1948). Since then, psychophysics has been used as a methodology to investigate the relationship between the objective, physical elements of the world and their subjective, psychological percepts. When studying perception, through psychophysics neuroscientists have been able to evaluate sensory thresholds (both absolute and differential) and investigate various neural mechanisms without resorting to invasive methods (Anobile et al., 2016, 2020; Binda et al., 2009; Domenici et al., 2021a, 2021b; Fornaciai et al., 2016; Togoli et al., 2021; Tonelli et al., 2017, 2020). This evaluation requires the presentation of stimuli close to participants’ sensory thresholds so that intensity differences between stimuli are often small scale. Thereby, stimuli must often be delivered with high-temporal resolutions, and particular care is taken when presenting stimuli that need to be as precise (i.e., stable across time) and accurate (i.e., close to the desired intensity) as possible.

Due to these technical requirements, psychophysical stimuli must be both accurate and precise in terms of onset, offset, and duration, making online testing sometimes tricky to perform. Nonetheless, in recent years many platforms devoted to remotely collecting data have been created (Giamattei et al., 2020; Stoet, 2017). For instance, as early as 2017, Finger and colleagues (Finger et al., 2017) developed LabVanced (labvanced.com), a JavaScript web application that includes an online editor suitable for developing behavioral experiments. Notably, LabVanced shows extreme accuracy in collecting online reaction times (Lukács & Gartus, 2023) as well as good timing performance when delivering visual stimuli within the 1000–1100-ms range. Similarly, Anwyl-Irvine and colleagues (Anwyl-Irvine et al., 2020) recently created another online builder, namely Gorilla Experiment Builder (gorilla.sc), through which it is possible to design and perform behavioral experiments via online testing. While both builders represent reliable and easy-to-use tools to perform online data collection, Pronk and colleagues (Pronk et al., 2020) showed that both reaction time evaluations and stimuli durations are affected by the operative system installed on the machine, as well as by the browser running the experiment. Furthermore, online platforms can provide precise visual and auditory stimulations, but they inherently lack the possibility to involve the tactile sense in a similar fashion.

In light of Pronk and colleagues’ results, it should be noted then that the risk of measurement biases within results obtained through online testing is always lurking. This is particularly relevant as each participant naturally performs experiments using their own hardware and software, with the additional confound of the browser used to run the experiment that might differ as well across users. Involving the same experimental setup when collecting data might be preferred, although sometimes not practical (e.g., during the recent COVID-19 outbreak, when recruiting participants to research facilities – where high-performant setups are held – was almost impossible). Additionally, collecting adequate amounts of data is always a relevant issue for behavioral research: this is crucial in discovery studies, in which statistical significance is compared against a fixed threshold and underpowered designs result in inflated effect sizes (Ioannidis, 2008). Moreover, both sample sizes and number of observations drastically influence the occurrence of type 1 error, i.e., accepting false-positive results when comparing them against the null hypothesis (Simmons et al., 2011). Collecting adequate sample sizes in one single batch should also be preferred, as adding data to an already-collected dataset increases false-positive rates as well, especially when statistical significance is close to the threshold (Murayama et al., 2014).

Thus, it is not surprising that many researchers have recently managed to develop portable solutions to foster data collection on a remote, broader scale. Bignardi and colleagues, for example, developed RED (Resilience in Education and Development), an app for tablets suitable for performing batteries of cognitive tests in primary school children (Bignardi et al., 2021). In a similar fashion, Bhavnani and colleagues developed DEEP (DEvelopemntal assessment on an E-Platform), an Android app focused on evaluating cognitive abilities in children as young as 3 years of age (Bhavnani et al., 2021). Additional technological solutions have also been proposed in precedent years (Pitchford & Outhwaite, 2016; Robinson & Brewer, 2016), supported by the fact that cognitive evaluations collected via remote (e.g., tablets) are equally reliable to canonical data collected in a laboratory setting (Semmelmann et al., 2016). Despite this, most of the apps developed focused on the evaluation of cognitive functions, which in turn requires lower temporal precision than psychophysical investigations, where accuracy of stimulus timing is paramount.

Thereby, we started figuring out a solution to perform psychophysical testing directly at participants' homes and, potentially, rehabilitation centers. Thus, we designed and developed *PsySuite*, an Android app aimed at setting psychophysics to a portable level. We opted for the involvement of smartphones since, nowadays, such devices are affordable and highly user-friendly. Smartphones are also well suited for delivering stimuli in the visual, auditory, and tactile modality, simply displaying images on the screen, reproducing audio tracks, or delivering vibrations across the case. Notably, smartphones have already been used to foster a remote approach to psychophysical testing: recently, Marin-Campos and colleagues developed StimuliApp, an application developed on iOS that can be used to generate visual and auditory psychophysical tests via pre-selected templates (Marin-Campos et al., 2021). While StimuliApp allows for better customization on the user’s end, being originally developed for iOS its involvement is inevitably more expensive due to the price range of Apple smartphones. Furthermore, StimuliApp cannot deliver tactile stimuli, and might be impractical for generating multimodal stimulations.

To assess PsySuite’s feasibility in collecting reliable psychophysical measurements, we aimed to validate the app considering both its hardware performance and overall ability to replicate typical perceptual phenomena elicited in canonical settings. Heretofore, *PsySuite* already underwent two preliminary validations (Domenici, Inuggi et al., 2021, 2021). In the present study, one of our goals was to expand previous validation routines by systematically quantifying the stimulus timing via an external oscilloscope. This validation process, from now on referred to as hardware validation, focused on testing whether the app could deliver accurate and precise stimuli across sustained solicitations. For completion purposes, hardware validation was performed on two different midrange smartphone models: a Xiaomi Mi A2 and a Samsung A40. Importantly, since multimodal synchrony was dependent on the smartphones’ models involved, we suggest the reader focus on the Psychophysical Stimuli’s Results section before using the app. In order to be suitable for psychophysical testing, we expect that PsySuite can produce any possible combination of visual, auditory, and tactile stimulations with sufficient precision and accuracy, even when significantly stressed due to prolonged use. Ensuring that PsySuite correctly reproduces various durations across different timescales is necessary, but not sufficient, to foster its involvement in psychophysical research. In fact, it is also relevant to prove that behavioral measurements obtained with the app are equivalent to the ones obtained via the more classical PC-based setups. Therefore, we performed a second validation routine, from now on referred to as behavioral validation, comparing participants' performance in a classic psychophysical task developed within PsySuite with the performance in the same task developed using a classic PC-based setup.

In the end, *PsySuite* was designed and developed considering the following peculiarities:

- Deliver accurate and precise stimulations in visual, auditory, and tactile modality;
- Make psychophysical testing accessible, allowing participants to perform experimental sessions in complete autonomy (including participants with sensory disabilities);
- Send experimental results by e-mail at the end of the procedure;
- Make experimental procedures interruptible and resumable.

### PsySuite’s interface

*PsySuite*’s interface allows the user to select different tests from the very first screen. In addition to an ensemble of classical psychophysical paradigms (Engel & Singer, 2001; Gori et al., 2012; Rammsayer & Lima, 1991; Shams et al., 2000), the app is also equipped with a module suited to generate a wide variety of uni- and multi-modal stimulations. The latter tool has proven to be crucial during the validation of the hardware performance, and, together with the behavioral test used for the validation of the psychophysical results, has thus been included in the modules available for administration.

**Fig. 1:**
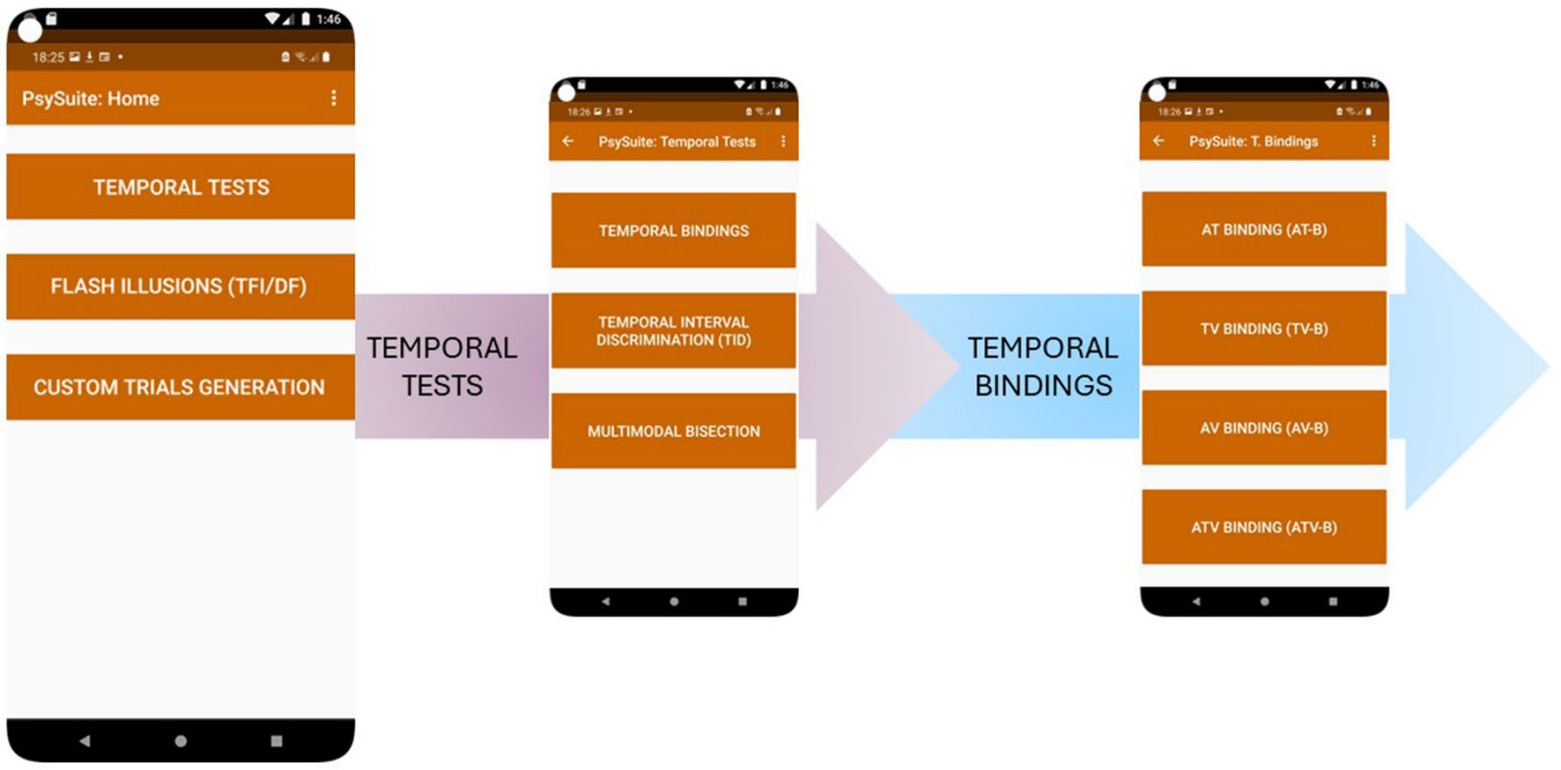
*PsySuite*’s home screen. By selecting one of the three possible choices, further screens are provided to make the selection of the task as intuitive as possible. In the current figure, the user is selecting the “TEMPORAL TESTS” button, which leads to the three different tests available. Once the user selects the task of interest (in this case, temporal bindings), a last screen appears for the selection of the sensory modalities

The home screen is shown in Fig. 1. From there, the generative tool can be accessed by selecting the last button, labeled “CUSTOM TRIAL GENERATION”. This module was used for the app’s instrumental validation and allows the generation of an ensemble of uni- and multi-modal stimulations delivered in succession. From the initial interface of the module, the user can select the experimental condition and parameters of interest, so to generate a continuous stream of stimuli and evaluate the device’s stability across the whole sequence (when measuring it via an external oscilloscope). For additional information about the parameters to be defined for this procedure, the user can access *PsySuite*’s manual by pressing the three vertical dots in the top-right corner of the home page, as well as at the following link: https://gitlab.iit.it/u-vip_public/psysuite/app/-/blo/blob/master/psysuite_reviewers_manual.

**Fig. 2:**
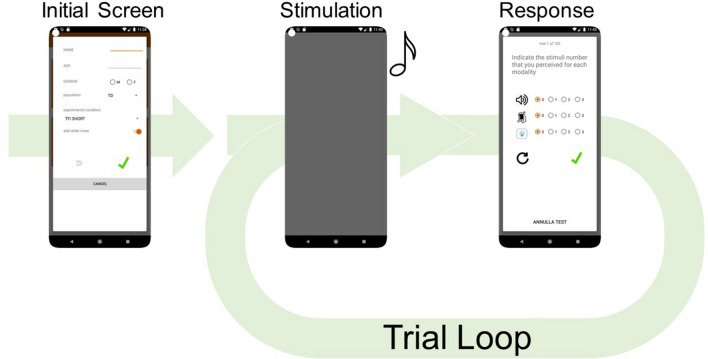
Initial screen and trial loop for all experiments implemented within *PsySuite*. In the initial screen, the participant can indicate their name/ID, age, gender, and the population of reference (by default, TD = typical development). According to the experiment, different conditions can be selected (please, refer to the user’s manual for an exhaustive description of all procedures). To mask the noise produced by the case vibration, additional white noise can be played in the background. Once the participant presses the green checkmark, the trial loop starts with each trial being developed as follows: first, the stimulation is delivered (in this sample case, one auditory stimulation is presented). Then, the response screen appears, and the participant must indicate how many stimuli they perceived. Once the green checkmark is pressed, the next trial starts. The *black arrow* near the *green checkmark* can be used to replay the trial, in case the participant missed the presentation of the stimulus/i

Going back to the main screen, the user can select two additional buttons: “TEMPORAL TESTS” and “FLASH ILLUSION (DFI/TFI)”. Here, we will briefly introduce all experimental designs included in *PsySuite*. However, for a comprehensive explanation, we recommend once again referring to the manual mentioned above. Figure 2 represents the trial loop, which was common to all experimental designs.

### Temporal tests

The “TEMPORAL TESTS” button leads to a different screen, from which the user can choose between three different tests.

### Temporal bindings

Selecting the “TEMPORAL BINDINGS” button will open a third, final screen from which the user can select the sensory modality involved. Temporal binding tasks required participants to report whether two (or more) stimuli are synchronous or separated by a given temporal interval. Within *PsySuite*, temporal bindings can involve all possible bimodal combinations, as well as all three sensory modalities simultaneously.

### Temporal interval discrimination (TID)

The “TEMPORAL INTERVAL DISCRIMINATION (TID)” button leads to a temporal interval discrimination task, in which participants have to indicate which one of two intervals delivered in sequence lasts longer. Intervals to be judged are delimited by uni-sensory stimulations that can be either sounds, phone vibrations, or flashes displayed, and do not change across conditions (meaning that an interval delimited by sounds will always be compared against another interval delimited by sounds; the same goes for phone vibrations and visual flashes).

### Multimodal bisection

Following the “MULTIMODAL BISECTION” button, the user gains access to a specific version of the temporal bisection task. In the bimodal bisection task, three stimuli are delivered in sequence, separated by a given intervals. The participants’ task is to report whether the second stimulus of the sequence was closer in time to the first or to the third, virtually bisecting the overall interval represented by the first and last element of the sequence.

### Flash illusion (DFI/TFI)

The flash illusion is a notorious perceptual effect that arises when presenting an incongruent number of multi-modal stimulations (e.g., two beeps and one flash) and asking participants to indicate how many flashes they perceived. If delivered correctly, participants will report a higher number of flashes compared to the true number of presented visual stimuli. As this test was used for the validation at the behavioral level, its discussion is further extended in the corresponding paragraph below.

#### Architecture and implementation

*PsySuite* was developed within the Android environment as Android smartphones have broader market availability and reduced cost compared to other smartphone brands. Since the app was designed to sustain performance-demanding data collection procedures, we targeted modern smartphones equipped with the most recent versions of Android OS. *PsySuite* was compiled considering an Android SDK with a level of at least 27 (corresponding to the Android 8.1 version). We used Kotlin as the programming language and AndroidX as a library package. Since Kotlin is an infrequent programming language, we added a step-by-step tutorial on how to create a simple reaction time task in the Supplementary Information.

### Stimuli generation process

Undeniably, the stimuli generation procedure is the core element of the whole app. Psychophysical research involves uni- and multimodal stimulations that must be conveyed using accurate and precise timing. Specifically, using *PsySuite,* we aimed to deliver reliable visual, auditory, and tactile stimulations, both individually and in any possible combinations.

Acoustic stimuli have proven to be the most problematic to generate among all sensory modalities involved because the Android Audio Framework is composed of several components that work together, even though they do not guarantee complete determinateness of output latencies (Gokul et al., 2016). The out-of-the-box audio solution developed within Android environment relies on the MediaPlayer API, which is a high-level solution able to play back various compressed audio formats, even though it is difficult to produce low-latency output streams following this interface. Thereby, we decided to explore two different, potentially more accurate solutions: the AudioTrack Java API and a C +  + native solution executed via the OBOE library (https://github.com/google/oboe), which inherently support the development of high-performance audio app on Android OS. We sampled auditory stimuli considering the system's optimal sampling frequency to implement low-latency playback audio streams, encoding them in an uncompressed format. Therefore, audio stimuli were stored in an uncompressed 16-bit PCM format with a sampling rate of 48 kHz and saved as.WAV files. At the beginning of each experimental session, recorded stimuli were preloaded in a dedicated audio buffer and repeatedly played, reducing timing latencies as much as possible. Audio resources were released at the end of each experimental session to generate stimulations.

AudioTrack is a low-level Java API mapping directly into the native code, allowing the reading of each.WAV file's content as ByteArray and copying it into their own object's buffers (ran in static mode). The playback head position is fixed at zero within the static buffer at the end of each auditory presentation.

Conversely, the OBOE library is a Google C +  + library developed around *ESAudio* (if the SDK level is less than 26) or *AAudio* (if the SDK level is equal to or higher than 27), proposing several optimization routines to reduce input/output timing latencies significantly. Both AudioTrack and the native OBOE solutions were initially considered when producing auditory stimuli, and even though, in the end, the latter was chosen due to its more straightforward implementation and superior reliability.

Visual stimuli were created by storing 8-bit PNG drawables within an ImageView component located in a Fragment at the start of each experimental session. Onsets, offsets, and duration of the stimuli were controlled by modifying the component's visibility.

Tactile stimuli were implemented using Android's VIBRATOR_SERVICE, and modeled using the vibrate and cancel methods of the Vibrator inherent class.

#### Hardware validation

To validate *PsySuite*'s performance, we ran technical measurements on two different smartphones: a Xiaomi Mi A2, with 3 GB of RAM and a Qualcomm® Snapdragon™ 625 Octa-Core processor running at 2.0 GHz, and a Samsung A40, with 4 GB of RAM and an Octa-Core processor (2 × 1.8-GHz Cortex-A73 and 6 × 1.6-GHz Cortex-A53). Android v. 10 was installed as an operating system in both smartphones, and both smartphones' refresh rate was 60 Hz.

To measure all different signals produced by *PsySuite*, we used a TDS 2014B Tektronix oscilloscope. Visual stimuli were measured through a phototransistor cabled to an external amplifier set at 5 V. Tactile stimuli were measured using a brass disk-shaped piezoelectric sensor with a diameter of 27 mm and a thickness of 0.4 mm. Auditory stimuli were measured by acquiring the signal directly from the smartphones' 3.5" jack output. All sensors were simultaneously connected via crocodile cables to the oscilloscope across different channels.

The oscilloscope was directly plugged via USB cable to a Lenovo X220 notebook, running MATLAB v. 2013. Each signal was stored within a structure and then evaluated using MATLAB custom-made scripting procedures through post-processing signal analyses. Regardless of the sensory modalities involved, signals were recorded with a 10,000-Hz sampling frequency and measured within a timeframe of ± 125 ms (with 0 being the approximate stimulus' onset by definition), thereby allowing the recording of oscilloscope traces both before and after stimuli delivery.

During post-processing analyses, auditory and tactile signals were smoothed using a Savitzky–Golay filter, fitting subsequent sets of signals' adjacent points with a low-degree polynomial and the linear least-squares method. We scanned for abrupt changes in the signals' traces to mark stimuli's onsets and offsets. Thereby, we evaluated the signals' medians and standard deviations before stimuli delivery and considered stimuli's onsets when traces suddenly exceeded a given cutoff, fixed at the averaged signal's value before the stimulus' onset ± two standard deviations. Using a similar procedure, we evaluated the last traces' points in which the signal exceeded the same cutoff value so that we were able to pinpoint stimuli's offsets as well. Visual signals were smoothed using a low-pass filter with a normalized passband filter of 0.1 π rad/sample. We divided the oscilloscope's traces into different partitions to identify abrupt changes. We evaluated the point that minimized the sum of the residual squared error from its local averaged value for each partition. Setting the number of regions as a free-to-vary parameter, we could locate all the significant signals' frames denoting stimuli's onset and offset, i.e., frames in which the signal significantly changed. For all sensory modalities, stimuli durations were evaluated simply by calculating the time difference between stimuli's offset and onset.

### AudioTrack vs. OBOE solution

**Fig. 3:**
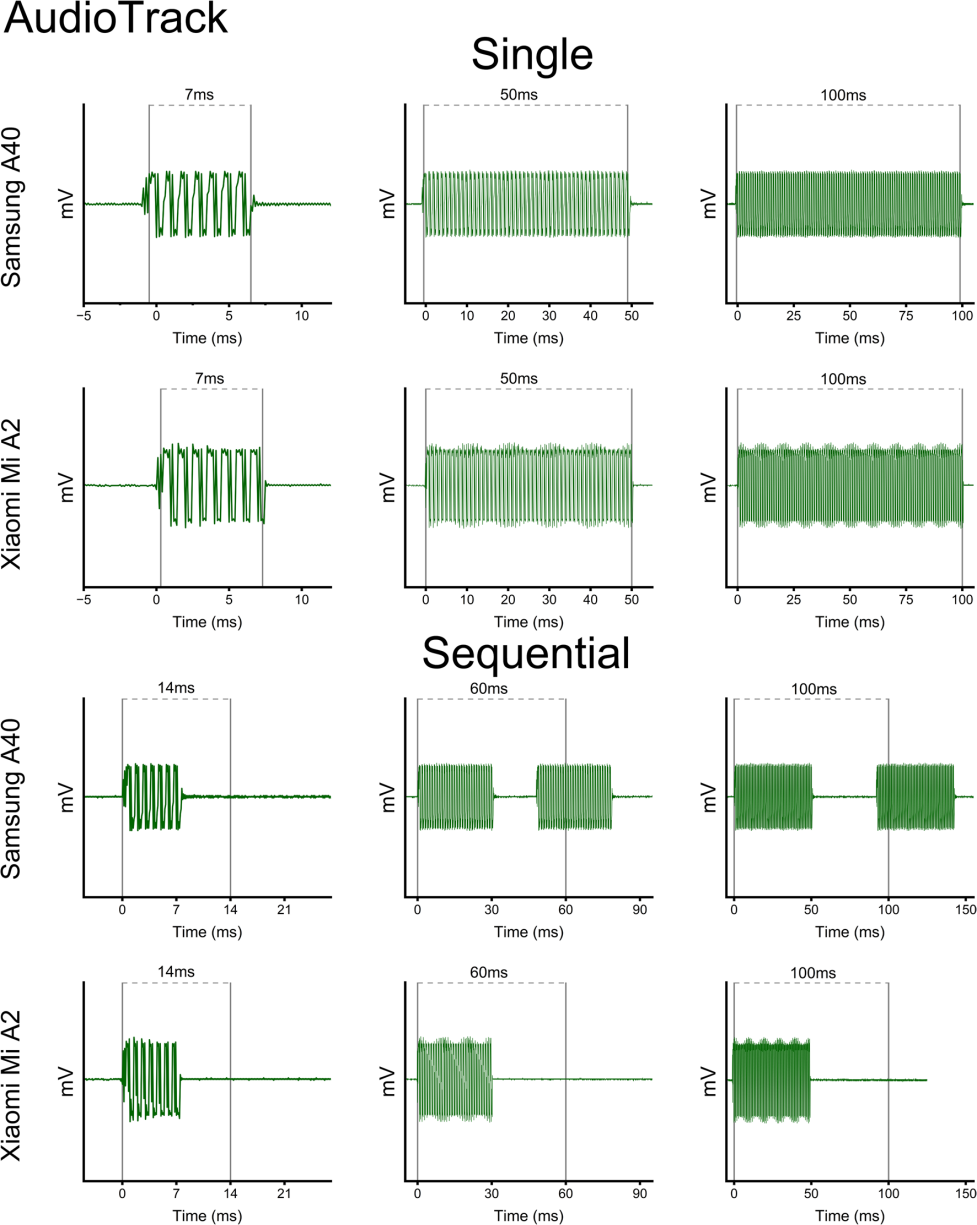
AudioTrack auditory stimuli generation. Traces of the auditory stimuli produced through the AudioTrack solution, using both the Xiaomi MI A2 and the Samsung A40 smartphones. As one can appreciate, AudioTrack failed to generate appropriate auditory signals – even fizzling to produce pairs of stimulations when the two stimuli were separated by shorter intervals for both smartphone models. *Vertical bars* indicate expected stimuli onsets and offsets in the single condition, when only one stimulus was delivered, and expected onsets for both stimuli in the sequential condition, when two stimuli were delivered in succession

**Fig. 4:**
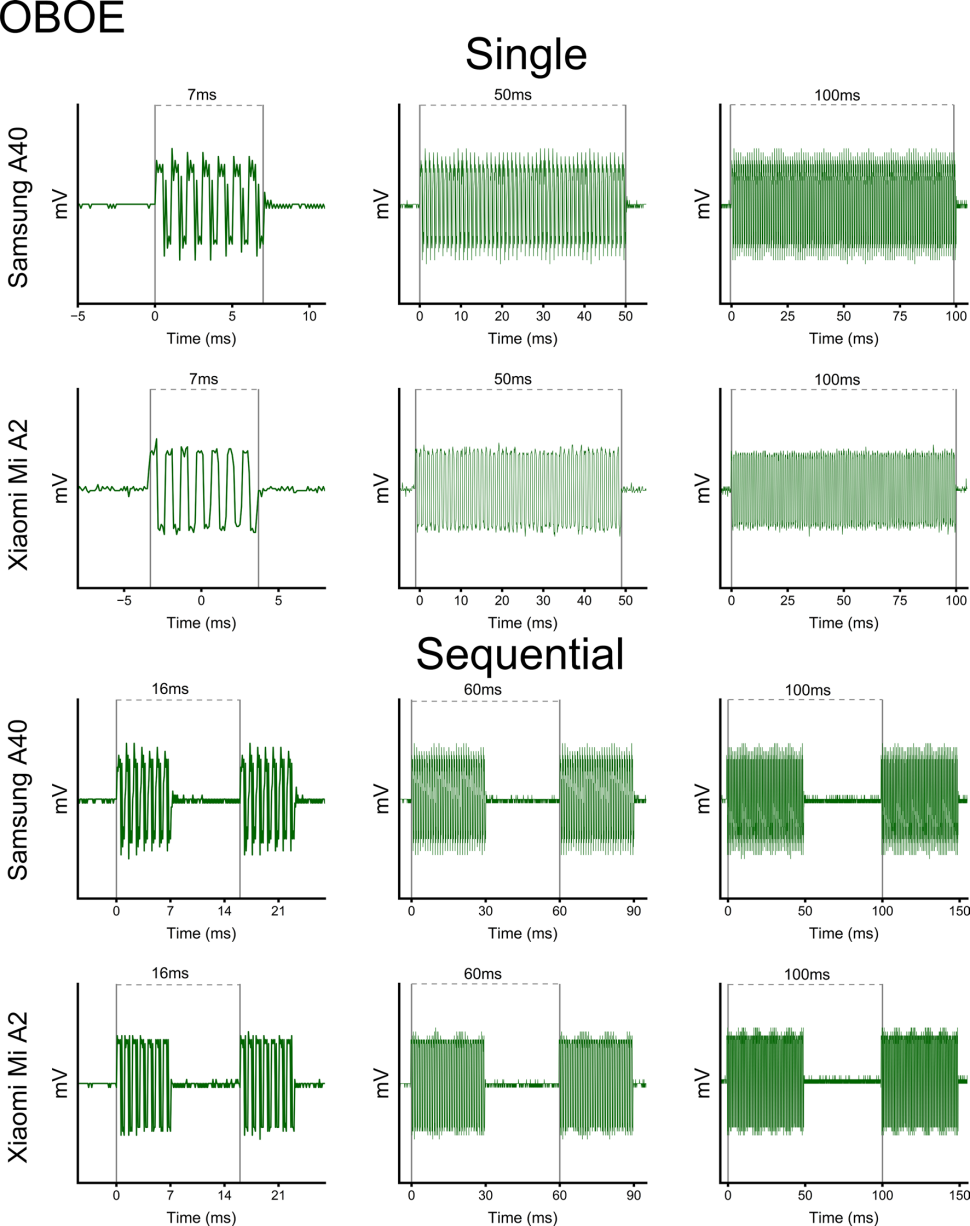
OBOE auditory stimuli generation. Traces of the auditory stimuli produced through the OBOE solution, using both the Xiaomi MI A2 and the Samsung A40 smartphones. Conversely to the AudioTrack solution, OBOE managed to generate appropriate auditory signals in virtually all conditions but the shortest interval production, which was overshot by 2 ms. *Vertical bars* indicate stimuli onsets and offsets in the single condition, when only one stimulus was delivered, and onsets for both stimuli in the sequential condition when two stimuli were delivered in succession

To implement the best overall solution in delivering accurate and precise auditory signals, we compared the stimuli-generation procedure of both smartphones when using either the AudioTrack Java API or the OBOE native solution. We thus considered the performance of *PsySuite* when delivering different sets of stimuli configurations. To cover a wide range of possible scenarios, we compared the stability of the app’s signals when producing either individual or sequential stimulations. First, we generated single auditory stimulations lasting 7, 50, and 100 ms using both the AudioTrack API (Fig. 3, upper panels) and the OBOE native implementation (Fig. 4, upper panel). We then proceeded to test the app’s performance when generating two sequential simulations with similar duration, separated by a fixed interval. Specifically, we tested pairs of 7-, 30-, and 50-m stimuli separated by 14, 60, and 100 ms, respectively, testing once again both solutions (Figs. 3 and 4, lower panels).

Notably, we found significant differences between the AudioTrack Java API and native OBOE implementations. On the one hand, the OBOE native solution successfully produced all auditory stimuli (Fig. 4, upper panels), as well as all inter-stimuli intervals (Fig. 4, lower panels). Interestingly, both the Xiaomi MI A2 and the Samsung A40 achieved good performance capabilities when using the OBOE solution, although the shortest interval was systematically overproduced by 2 ms (see also Fig. 8). On the other hand, the AudioTrack Java API showed lower control over the generation of proper auditory stimuli. While all single stimuli were produced with high accuracy (Fig. 3, upper panels), using AudioTrack to generate sequential auditory stimulations were particularly problematic (Fig. 3, lower panels). Specifically, the Samsung A40 failed to produce pairs of auditory stimuli when the inter-stimuli intervals were shorter (i.e., 14 ms), and unreliably produced inter-stimuli intervals (often resulting in intervals shorter than the expected output). The Xiaomi MI A2 performed even worse when using the AudioTrack solution, as it failed to produce pairs of auditory stimuli even when intervals were supposed to last 100 ms. Given these results, for further auditory implementation we opted for the OBOE native solution, which was the preferred routine used to generate auditory stimuli.

### Psychophysical stimuli

To assess *PsySuite*'s feasibility in performing psychophysical experiments, we investigated its accuracy and precision in delivering uni- and multimodal stimulations. We performed different recording sessions consisting of 100 subsequent trials for all stimuli durations and sensory modalities combinations.

For each sensory modality or each of their possible combination, we tested and recorded with the oscilloscope the following stimulus durations: 7, 17, 30, 50, and 100 ms. Then, the app's accuracy was tested by evaluating how the produced stimulations matched their corresponding input parameters, i.e., whether onset, offset, and duration respected the values chosen by the experimenter. To obtain an accuracy index, we calculated the median of measured durations across each recording session. The app's precision was evaluated afterwards, and the accuracy of the stimulations was tested across prolonged experimental sessions. We thereby calculated the standard deviation of all 100 stimulations measured in each recording session while expecting reduced stimulations' variability if *PsySuite* maintained adequate stability over the generation procedure. Lastly, we evaluated the minimum temporal distance (i.e., the inter-stimulus interval, ISI) achievable between two stimuli generated with *PsySuite*. To develop proper ISI during recording procedures, we considered the minimum stimulus duration correctly reproduced in the previous tests for each sensory modality. For each possible duration, we applied an ISI that was double the minimum duration considered for that session and assessed whether the app managed to produce both stimuli in sequence. We started considering stimuli with a 7-ms duration (when *PsySuite* managed to produce stimuli as short as that) and an ISI of 14 ms. We then progressively increased both parameters according to the following formula:

1 $$ISI=2 \bullet stimulus\; duration$$

Concerning between-modalities simultaneity accuracy, we evaluated the reciprocal temporal shift considering sensory signals when all three modalities were involved. Such a shift is highly dependent on the hardware's and software’s intrinsic properties and cannot be changed. Thereby, we evaluated whether the stimuli's onsets were temporally misplaced and, eventually, corrected every single modality's onset so that they all occurred simultaneously. We reported all the results obtained after stressing the app with 100 trial-recording sessions on the following pages. When one of the smartphones failed to produce any stimulations (e.g., if the motor engines failed to turn on), we intentionally left the sample traces blank.

## Results

**Fig. 5:**
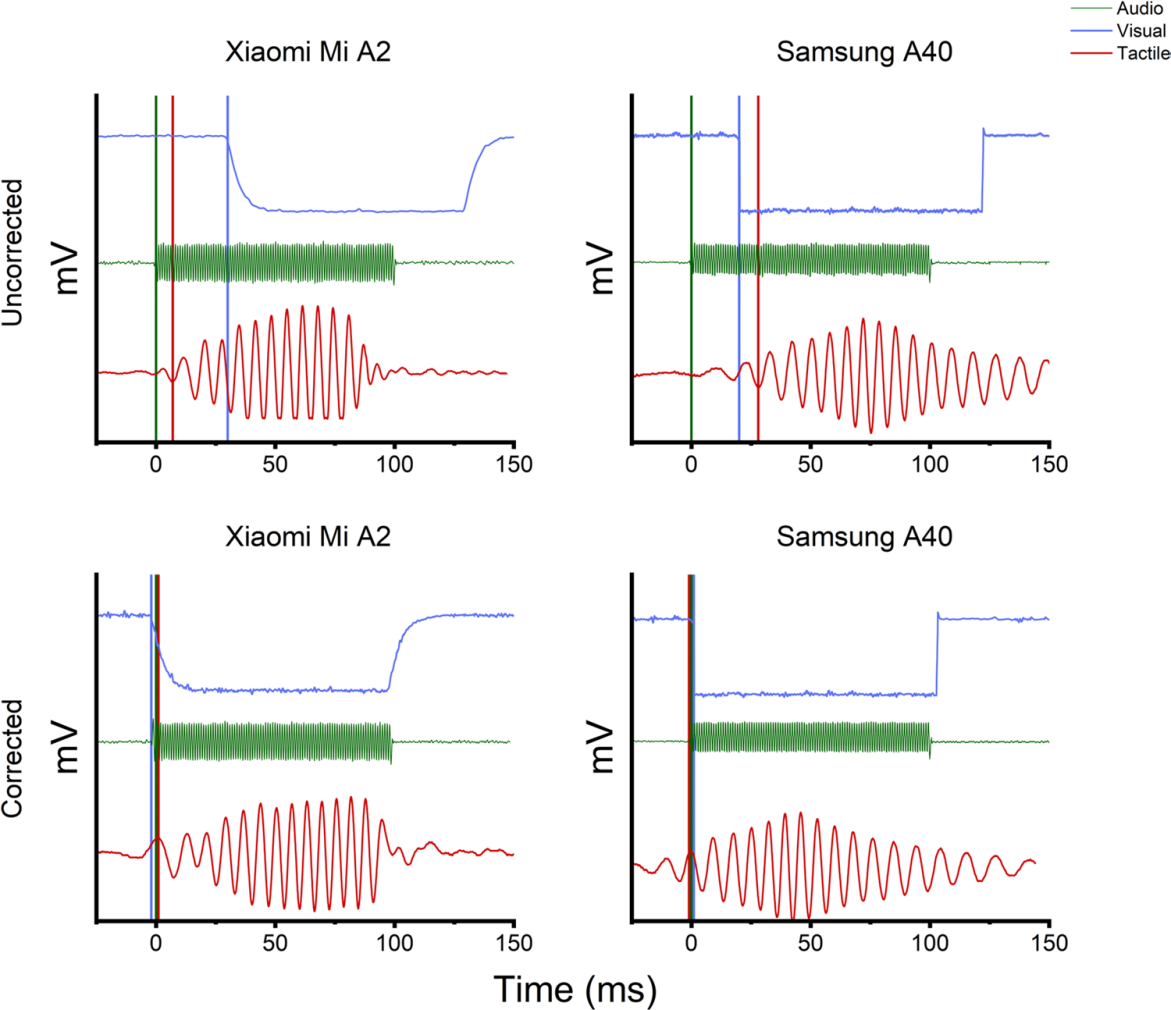
Uncorrected and corrected between-modalities simultaneity for both smartphones. Uncorrected (*upper panels*) and corrected (*lower panels*) between-modalities simultaneity for both smartphones. *Vertical colored lines* represent stimuli onsets for the corresponding color-coded modality

While unisensory stimulations obviously did not require any simultaneity between modalities, for multi-modal stimulations we first produced simultaneous tri-modal combinations and calculated the reciprocal shifts between auditory, visual, and tactile signals. By doing so, we were able to assess the between-modalities' simultaneity accuracy and, eventually, improve it. Overall, the Xiaomi vibrator's engine was much faster when compared to the Samsung one, even though tactile stimuli production was generally slightly slower than auditory stimuli production. Conversely, faster visual responses were observed using the Samsung smartphone, in line with previous measurements observed when delivering uni-modal stimuli. Samsung's faster performance in delivering visual stimulations resulted in shorter onset delays when compared to Xiaomi's. A slight temporal difference of ~ 5 ms could be observed when comparing visual signals produced by the two devices (Fig. 5, upper panels). Stimuli delays, with 0 being the faster (i.e., auditory) signals produced by definition, are reported in Table 1.

**Table 1:** Onset delays, expressed in milliseconds, measured delivering multi-modal stimuli with both smartphones. Positive numbers indicate that the corresponding stimulation began after the one for the quickest modality, which was always the auditory one

	Delay compared to auditory onset (ms)		
	Auditory	Tactile	Visual
Xiaomi MI A2	0	5	30
Samsung A40	0	28	20

Temporal delays were subsequently stored in a data structure and applied through an online run-time procedure executed during each trial. The module that managed stimuli delivery was equipped with a set of accessory functions able to identify modality combinations, calculate the specific delay among the onset of each stimulus, and then call the single primitive method. Such a method uses the Handler.postDelayed routine to enqueue single uni-modal calls in the UI thread's MessageQueue of the smartphone's OS. For example, assuming that the time difference between visual and auditory stimulations had a fixed delay of T_AV_, each time audio and visual stimuli are simultaneously displayed, the code started the first stimulus considering a delay of T_AV_ ms before delivering the second one, with the faster stimulus being delivered as first.

After applying adjustments based on the observed onset delays, simultaneous delivery of signals forming tri-modal stimulations was successfully generated (Fig. 5, lower panels). In light of these results, we would like to stress that while PsySuite is suitable to perform unimodal testing with whichever modern smartphone, multimodal testing requires planning from the user. Due to the inherent asynchrony of the visual, auditory, and tactile stimulations, when using a different model than the ones tested in this work, we strongly recommend re-testing the synchrony of all stimulations with an oscilloscope and, eventually, fixing it accordingly. Once all delays between modalities are noted, users can edit the proper config. file and re-build the app.

Once synchrony between stimulations was achieved, we assessed *PsySuite*'s feasibility in delivering stimuli suitable for psychophysical testing through systematic and accurate validation. Thus, we compared the technical performances of both a Xiaomi Mi A2 and a Samsung A40 devices, aiming to find the most appropriate smartphone to foster remote data collection procedures.

We considered three parameters for testing the app stability: the stimulations' accuracy, the stimulation's precision, and the minimum time difference between two stimuli sequentially delivered. An additional index, the between-modalities simultaneity accuracy, was initially evaluated to investigate whether the app could reliably produce appropriate simultaneous multimodal stimulations. We thus first measured the temporal difference between stimuli onsets when multimodal signals were displayed (Fig. 5, upper panels) and proceeded to correct the onset for each sensory modality (considering the onset of the auditory stimulation, the fastest one, as 0 by default) to achieve simultaneity of stimulation (Fig. 5, lower panels). The evaluation of this parameter was mandatory to deliver proper multimodal stimulations, and delays were easily fixed by introducing a temporal jitter to reduce the difference between onsets (Table 1).

### Single stimuli

Regarding auditory stimulations, no differences were found between the two smartphones. The OBOE native solution, which was ultimately chosen to deliver auditory stimulations, correctly played all.WAV files for their exact duration, and transitions from onsets and offsets were immediate and consistent with signal frequency. Notably, the AudioTrack Java API failed to produce sequential stimulations (up to the 30–60 couples, see Fig. 3, lower panels), being thus unreliable in delivering accurate and precise stimuli suitable for psychophysical testing.

For visual stimulations, the two smartphones' behavior was slightly different. Even though both devices could systematically reproduce durations from 17 to 100 ms, transition speeds were moderately dissimilar. The Samsung A40 smartphone was faster, with transitions within the order of 1 ms, while the Xiaomi Mi A2 showed slower transitions, up to 5 ms. Therefore, Samsung’s waveforms were closer to a square signal than those of Xiaomi. Unsurprisingly, durations shorter than 17 ms were not reproducible by any of the two smartphones due to the monitors' refresh rate, fixed at 60 Hz (allowing a minimum duration of 1/60 = 0.017 s). Further delving into the variance of visual signals, we also observed that both smartphones rarely misrepresented the selected duration by one frame (either reproducing one less or one more). Overall, the Xiaomi performed better compared to the Samsung, missing frame-perfect production only 8.25% of the time compared to the 12.75% measured with the Samsung A40. For the Xiaomi, this occurrence was more prominent for shorter durations (17 and 30 ms), for which perfect frame reproduction was missed around 12.5% of the time. However, for longer durations (50 and 100 ms) this percentage dropped significantly, with only 4% of total stimuli being one frame shorter or longer. Although visual signals might be less stable than ones produced via a desktop PC, these percentage are still way below what expected when using an Android OS to reproduce visual stimuli via different browsers, for which frame-perfect production of visual stimuli depends on the generation procedure used and peaks at around 82% (Pronk et al., 2020). Furthermore, perfect timing using online platforms is hardly achievable regardless of the OS (Anwyl-Irvine et al., 2021).

Lastly, when delivering tactile stimulations, smartphones' behavior was less reliable and significantly different overall. On the one hand, the Xiaomi Mi A2 managed to successfully produce tactile stimulations as short as 30 ms, showing a slightly lower precision when compared to the optimal values found in the other modalities. On the other hand, the Samsung A40 failed to reproduce accurate tactile stimulations, even when trying to deliver the longest stimulus possible (as setting an input duration of 100 ms led to vibrations lasting more than 125 ms). Waveforms produced by the Samsung device were significantly longer in all conditions (30, 50, and 100 ms), denoting an unstable control of vibrators engines. Altogether, the Xiaomi Mi A2 drastically outclassed the Samsung A40 in terms of both accuracy and precision. On a final note, tactile signal amplitude comparisons were challenging, as amplitude measurements depended on the sensor's position over the smartphone's casing. To overcome this technical issue, when performing psychophysical testing with *PsySuite* we asked participants to firmly hold the smartphone in their hand instead of letting them place one of their fingers over a specific smartphone's point.

### Sequential stimuli

When considering the minimum temporal difference achievable between two stimulations, similar patterns were highlighted considering the two smartphones, consistently with measurements obtained via single stimuli generation. Both devices appropriately performed when delivering auditory or visual stimulation pairs, although they delivered intervals slightly longer (of about 2 ms) when selecting the shortest ones. Furthermore, the Xiaomi Mi A2 drastically outclassed the Samsung A40 when producing pairs of tactile stimulations. While the former could reliably produce pairs of 30-ms tactile stimuli whose ISI was as short as 60 ms, the Samsung A40 always produced a single wave lasting about 125 ms. Consequently, the Samsung A40 device could not generate two distinct, separate waves when following the simple separation rule defined in Eq. (1), proving once more to be unfitting for psychophysical testing in the tactile domain.

**Fig. 6:**
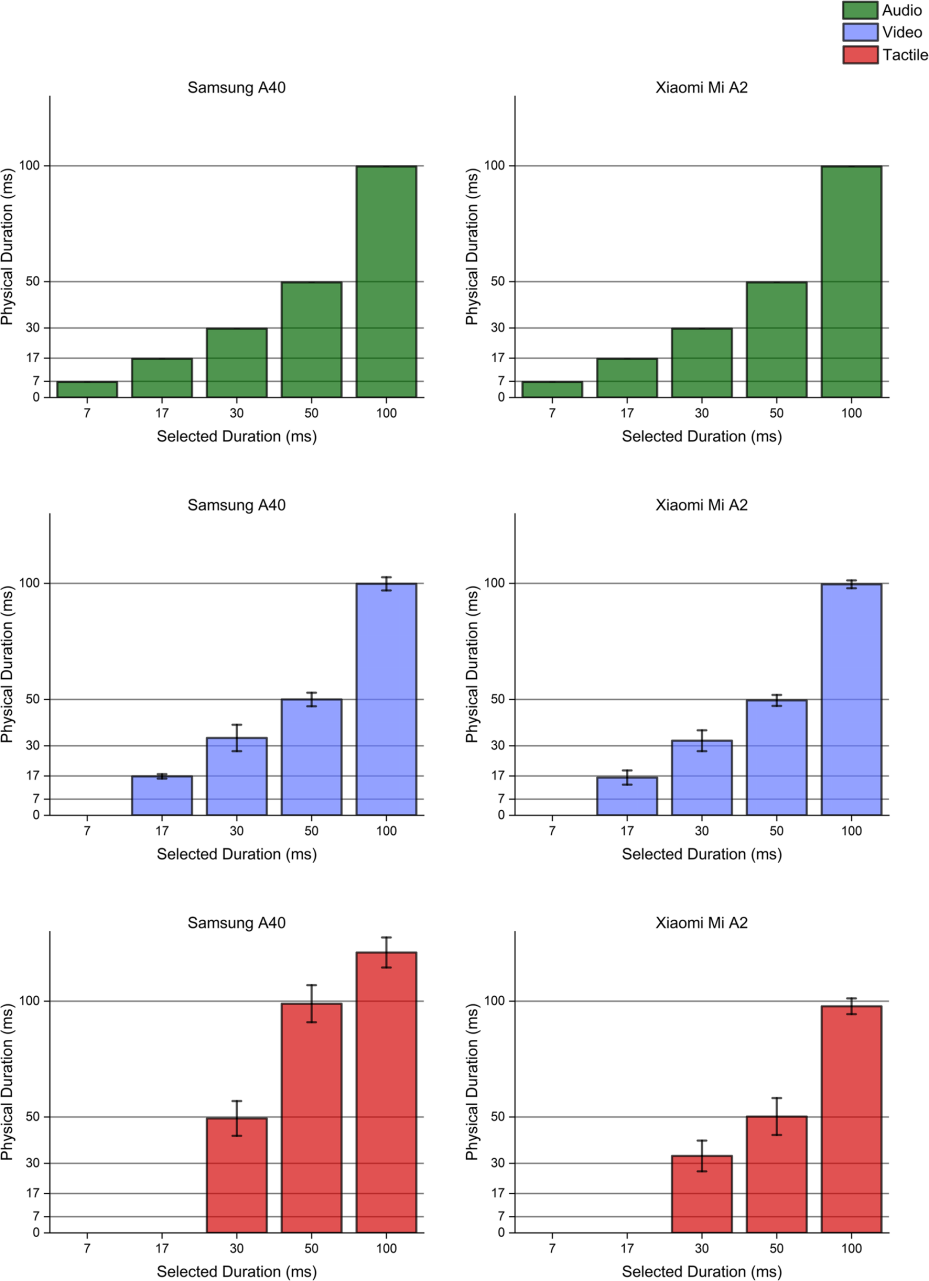
Unimodal single stimulations. Unimodal single stimulations delivered using either the Xiaomi Mi A2 or the Samsung A40. *Bars* indicate the medians for 100 repetitions of the stimulus duration (physical duration) related to the expected duration chosen by the experimenter (selected duration). *Error bars* indicate the mean absolute deviation measured for each set of repetitions. Auditory durations (*upper panels*, *green bars*) were reproduced with extreme accuracy and precision by both smartphones, as well as visual ones (*mid panels*, *blue bars*) which show little variability across presentations. Conversely, tactile durations (*lower panels*, *red bars*) showed a slightly higher variability when delivered using the Xiaomi Mi A2, while the Samsung A40 systematically delivered stimuli longer than expected. Note that for auditory stimuli the mean absolute deviation was extremely low (< 1 ms) so that error bars in the graph are virtually invisible

**Fig. 7:**
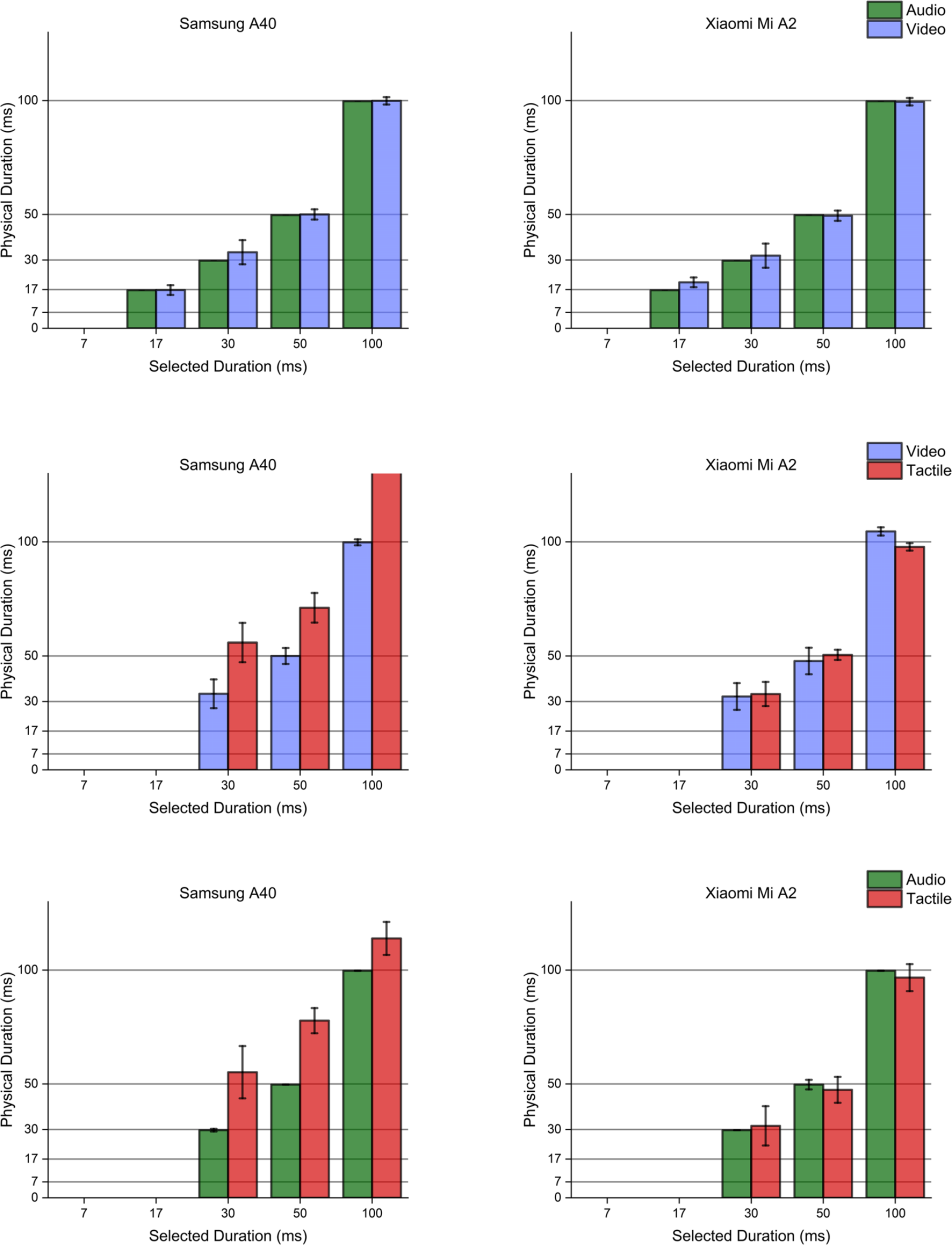
Bimodal single stimulations. Bimodal single stimulations delivered using either the Xiaomi Mi A2 or the Samsung A40. *Bars* indicate the medians for 100 repetitions of the stimulus duration (physical duration) related to the expected duration chosen by the experimenter (selected duration). *Error bars* indicate the mean absolute deviation measured for each set of repetitions. While the Xiaomi Mi A2 managed to produce accurate and precise bimodal stimulations in any of the possible sensory combinations, the Samsung A40 failed to reproduce accurate tactile signals, in line with stimuli generation patterns highlighted through unimodal stimuli delivery

**Fig. 8:**
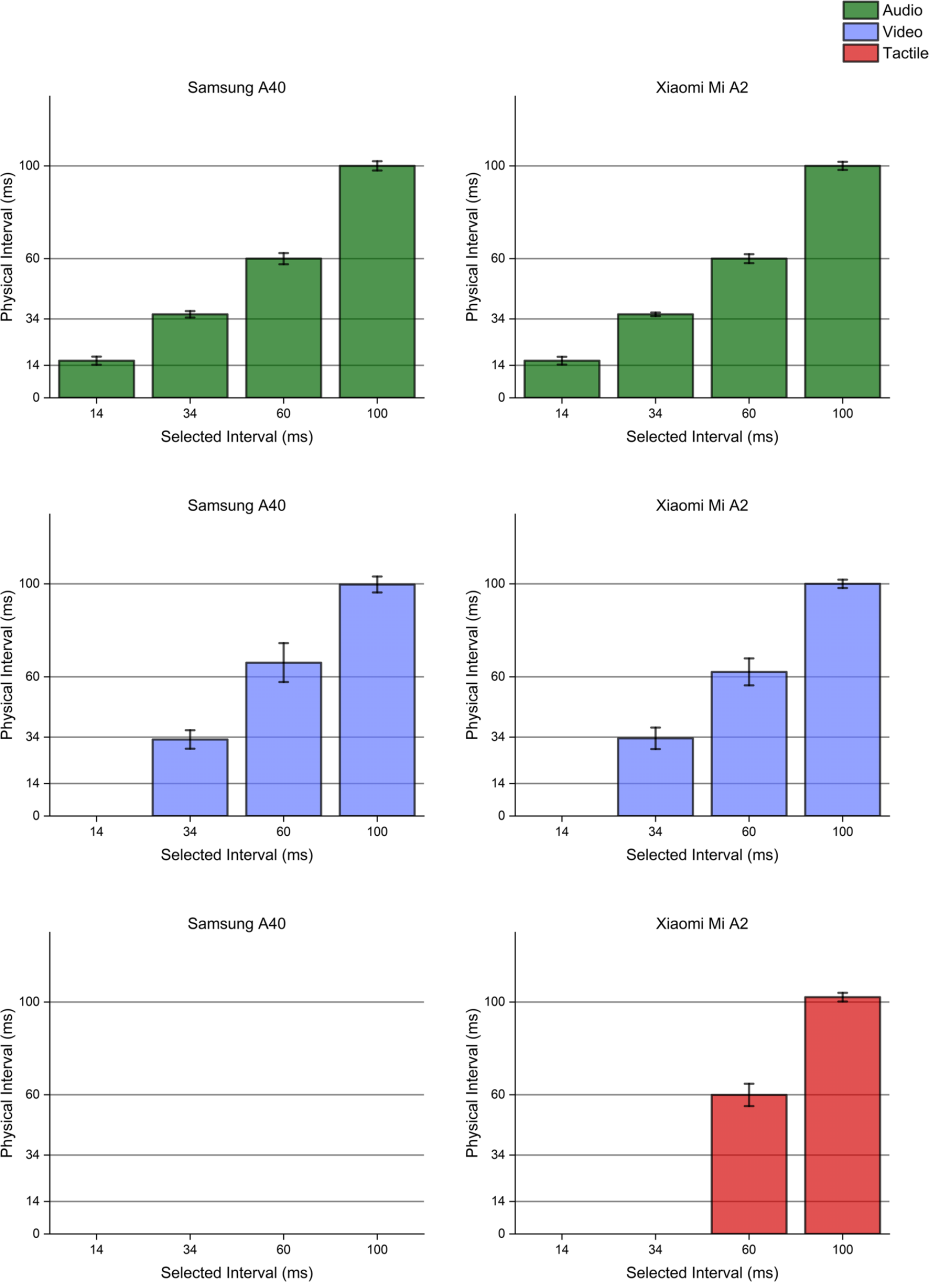
Unimodal sequential stimulations. Unimodal sequential stimulations delivered using either the Xiaomi Mi A2 or the Samsung A40. *Bars* indicate the medians for 100 repetitions of the interval between stimulations (interval duration) related to the expected interval chosen by the experimenter (selected duration). *Error bars* indicate the mean absolute deviation measured for each set of repetitions. Auditory intervals (*upper panels*, *green bars*) were reproduced with extreme accuracy and precision by both smartphones, while visual intervals were fairly more variable, especially when using the Samsung A40 (*mid panels*, *blue bars*). Conversely, tactile intervals (*lower panels*, *red bars*) were not reproducible with the Samsung A40, which just generated one single wave. The Xiaomi MI A2 definitely showed better performance, although it was able to generate intervals lasting at least 60 ms, failing to turn on the vibrator engines for lower intervals

**Fig. 9:**
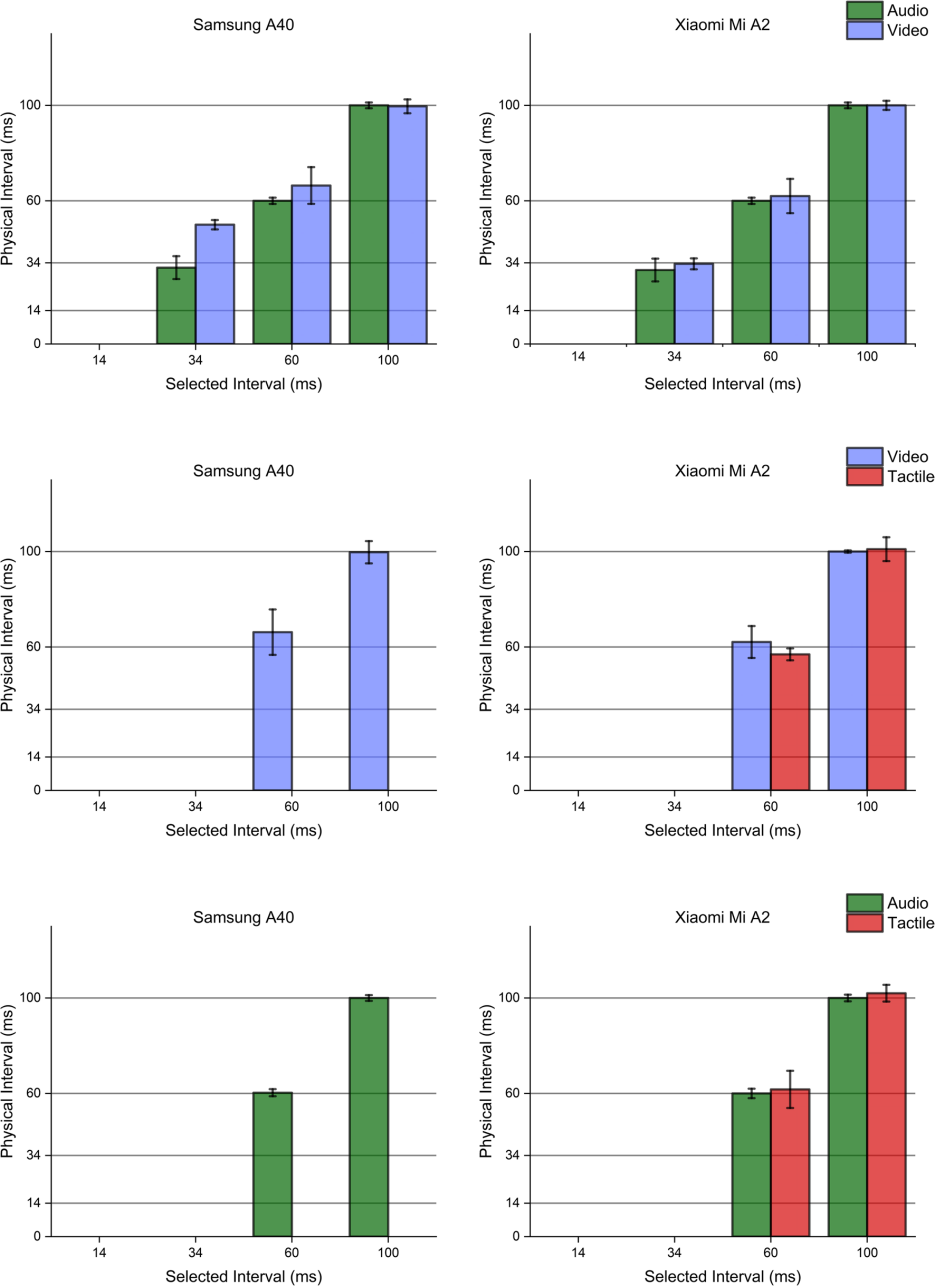
Bimodal sequential stimulations. Bimodal sequential stimulations delivered using either the Xiaomi Mi A2 or the Samsung A40. *Bars* indicate the medians for 100 repetitions of the interval between stimulations (physical interval) related to the expected interval chosen by the experimenter (selected interval). *Error bars* indicate the mean absolute deviation measured for each set of repetitions. While the Xiaomi Mi A2 managed to produce accurate and precise bimodal intervals in any of the possible sensory combinations, the Samsung A40 failed to reproduce any tactile interval, delivering a single wave lasting ~ 100 ms. These trends are, once more, in line with stimuli generation patterns highlighted through unimodal stimuli delivery

**Fig. 10:**
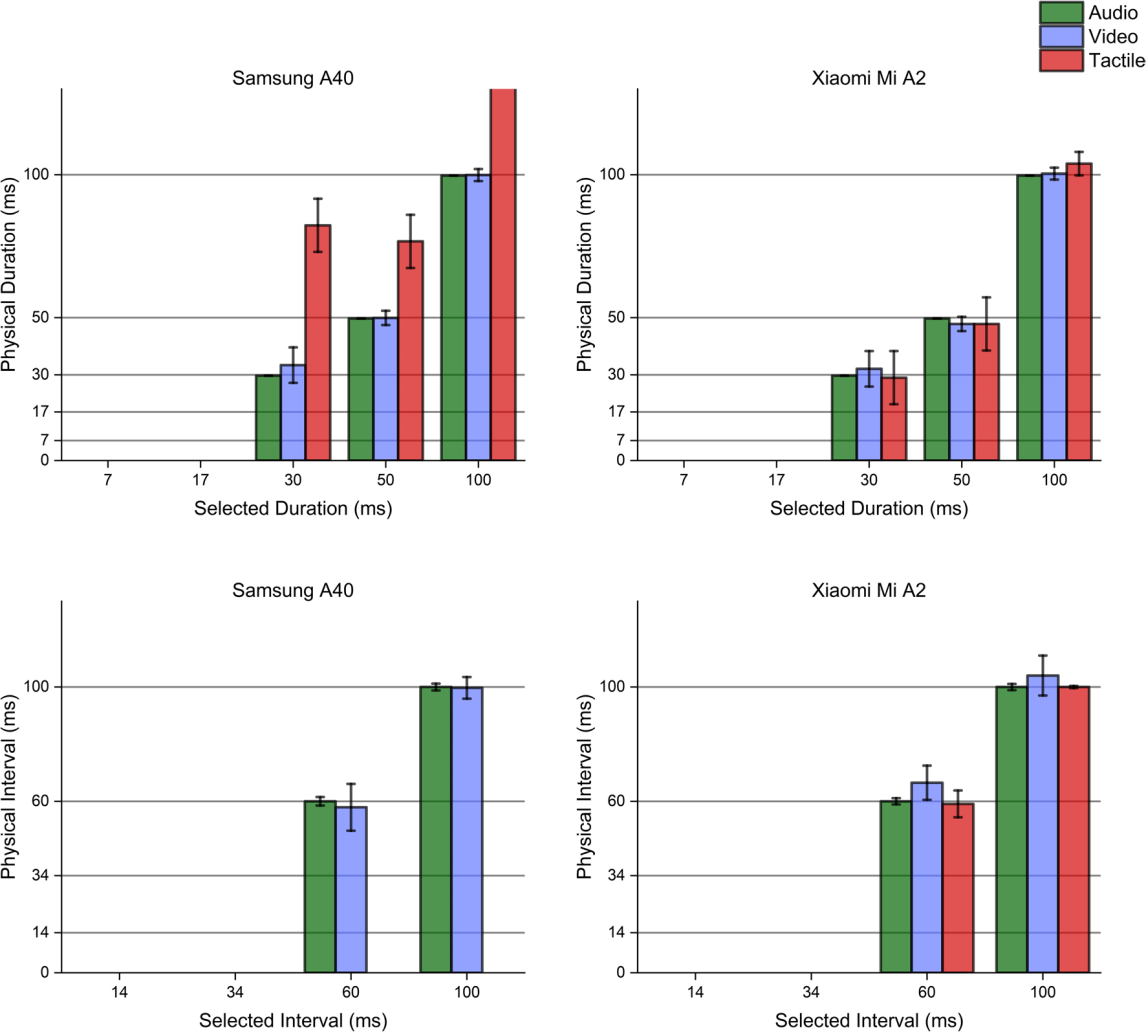
Trimodal stimulations. Trimodal single (*upper panels*) and sequential (*lower panels*) stimulations delivered using either the Xiaomi Mi A2 or the Samsung A40. *Bars* indicate the medians for 100 repetitions of the timing event of interest (physical duration/interval) related to the expected physical duration chosen by the experimenter (selected duration/interval). *Error bars* indicate the mean absolute deviation measured for each set of repetitions. While the Xiaomi Mi A2 managed to produce accurate and precise tri-modal stimuli and intervals in any of the possible sensory combinations, the Samsung A40 failed to accurately deliver tactile stimulations, as well as to reproduce any tactile interval producing a single wave lasting ~ 100 ms

Interestingly, multimodal stimuli generation procedures followed a trend similar to the one observed when producing uni-sensory stimulations. In all possible combinations of sensory modalities, the Xiaomi Mi A2 showed higher accuracy and precision than the Samsung A40, which failed to deliver vibrations of the proper duration. Even though the Samsung maintained slightly faster transition speeds for visual stimuli, its performance was generally less stable, becoming completely unreliable when tactile stimulations were involved. Hardware validation results are reported in Figs. 6, 7, 8, 9, 10.

Taken together, these results suggest that the Xiaomi Mi A2 was able to control the duration of the produced stimuli efficiently, regardless of the stimuli generated. At the same time, the Samsung A40 did not meet all the stability requirements to be suitable for remote data collection. Furthermore, stimuli generated with the Xiaomi Mi A2 were highly accurate and precisely reproduced, with the only limitations being tied to the refresh rate of the monitor (that did not allow the generation of visual stimuli shorter than 17 ms) and the slow response of the vibrator engines (that did not allow the generation of tactile stimuli shorter than 30 ms). Nonetheless, the lack of significant delays in stimuli reproduction and the overall solidity shown by *PsySuite* suggest that our app is potentially a befitting tool for remote psychophysical testing.

Since the Xiaomi Mi A2 drastically outclassed the Samsung A40 in terms of hardware performance and stimuli generation, only the former model was involved in all testing procedures developed after the hardware validation.

#### Behavioral validation

To ensure that data collected with a smartphone could be genuinely comparable to data obtained with classic setups, we developed a portable version of a double-flash illusion (DFI)-inducing experimental paradigm to assess whether psychophysical data collection could be efficiently performed using *PsySuite*. The DFI is a visual illusion occurring when a different number of auditory and visual stimuli are presented to an observer. In such a scenario, the influence of the auditory information determines the visual illusion, through which a different number of flashes is perceived. Presenting two auditory stimuli concurrently with one visual stimulus, for example, often leads to the illusion that two visual stimuli were instead displayed (Hirst et al., 2020; Keil, 2020; McCormick & Mamassian, 2008; Shams et al., 2000). Even though it has been demonstrated that touch can induce flash-illusions (Lange et al., 2011; Violentyev et al., 2005), to perform *PsySuite*'s behavioral validation, we focused only on sound-induced changes in visual perception.

Thus, we opted for implementing a DFI paradigm as the illusion is elicited only when stimuli durations and temporal displacements between stimuli are short enough (within the order of tens of milliseconds). High temporal resolution is required for the illusion to occur, and such resolution can be directly assessed by evaluating observers' performance (i.e., whether or not participants experience the visual illusion). Furthermore, *PsySuite*'s software and hardware components were highly stressed while performing the paradigm due to the strict time frames needed to elicit the visual illusion. Because of this, the DFI was considered an appropriate paradigm to evaluate the app's ability to perform reliable data collection procedures.

To test whether *PsySuite* could be a valuable tool to foster remote approaches to psychophysical research, we elicited DFI in participants directly using the app. Then, we compared the magnitude of the DFI obtained with *PsySuite* with the magnitude of a similar effect obtained with a more classic PC-based setup. If *PsySuite* can be successfully used to remotely collect behavioral parameters, we expect to witness two different results: first, we expect that observers experience the illusion when using the smartphone; second, we also expect the magnitude of the effects between the two setups to be comparable.

### Participants

A total of ten participants (four male, age, M = 30.4 years, SD = 5.4 years) were recruited to assess *PsySuite*'s capabilities in performing accurate behavioral testing. All participants had normal or corrected-to-normal vision and no history of neurological disease. All participants were naïve to the purpose of the study and gave informed consent prior to participation in the experimental procedure. Data collection was performed either at Genova's Istituto Italiano di Tecnologia or participants' homes when tested using *PsySuite*. Testing procedures were developed following the Declaration of Helsinki and were approved by the local ethical committee (Comitato Etico ASL3, Genova).

### Apparatus and stimuli

For the DFI elicited through *PsySuite*, stimuli are described in the Methods section. For the DFI elicited through the PC-based setup, stimuli were generated using the MSI caterpillar (Gori et al., 2019), a custom-made device plugged via a USB cable to a tower PC, directly controlled with MATLAB v. 2020b. The DFI task was coded on MATLAB as well (v. 2020b), being that the interface through which stimuli were generated and delivered (for additional information, please refer to the corresponding manuscript mentioned above). Such a device is tailored explicitly for multimodal stimulation, as it reliably delivers stimuli in the auditory, visual, and tactile modalities. During the procedure, participants sat at a 57-cm viewing distance from a 24" Asus VG248QE LCD monitor with a 1920 × 1080 pixels resolution and a refresh rate of 100 Hz. The monitor was used to display a red fixation point on an otherwise black screen, at which participants stared for the whole duration of the procedure, while the MSI caterpillar was placed two degrees of visual angle under it. Visual stimuli generated with the MSI caterpillar were 17-ms blue flashes subtending two degrees of visual angle, while auditory stimuli were 1-kHz pure tone bursts lasting 7 ms. To control for possible learning interference, the order of setups was counterbalanced across participants who performed either the task with *PsySuite* or with the PC-based setup at first.

### Experimental procedure

**Fig. 11:**
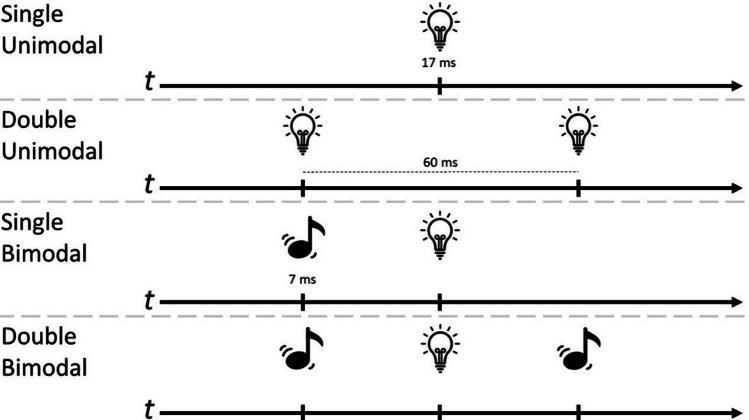
Trial classification for the DFI task. Different trial conditions developed to evaluate the DFI using both PsySuite and a PC-based setup

Similar experimental procedures were implemented using both *PsySuite* and the classic PC-based setup. Four different trial conditions were developed (Fig. 11): a set of trials in which one single visual stimulus was delivered (single uni-modal condition), a set of trials in which two visual stimuli were sequentially delivered (double uni-modal condition), a set of trials in which one visual and one auditory stimulus were delivered (single bi-modal condition), and a set of trials in which one visual stimulus and two auditory stimuli were delivered, i.e., the trial condition in which we would expect the illusion to occur (double bi-modal condition). Each type of trial was repeated 20 times, leading to a total of 80 trials that were presented to participants in random order. The delay between stimuli onsets was fixed at 30 ms so that the shortest kind of trial (single uni-modal) lasted 17 ms, and the longest kind of trial (double uni-modal/double bi-modal conditions) lasted 67 ms. At the end of each trial, participants reported how many visual flashes were displayed. Notably, the single unimodal condition was run only using the smartphone, while the double bimodal condition was run both on the smartphone and a classic PC-based setup.

### Data analysis

To assess *PsySuite*'s feasibility in performing efficient behavioral data collection, we first calculated the proportion of correct responses for both the single uni-modal and the double bi-modal condition obtained with *PsySuite* and the classic PC-based setup, respectively. Then, to evaluate whether we successfully elicited the DFI using *PsySuite* stimuli presentation, we compared proportions of correct responses between the two trials conditions of interest obtained when participants performed the task using the app. Lastly, to compare DFI's magnitude observed within the two different setups, we compared the proportion of correct responses in the double bi-modal condition measured using *PsySuite* and the classic PC-based setup.

### Results

**Fig. 12:**
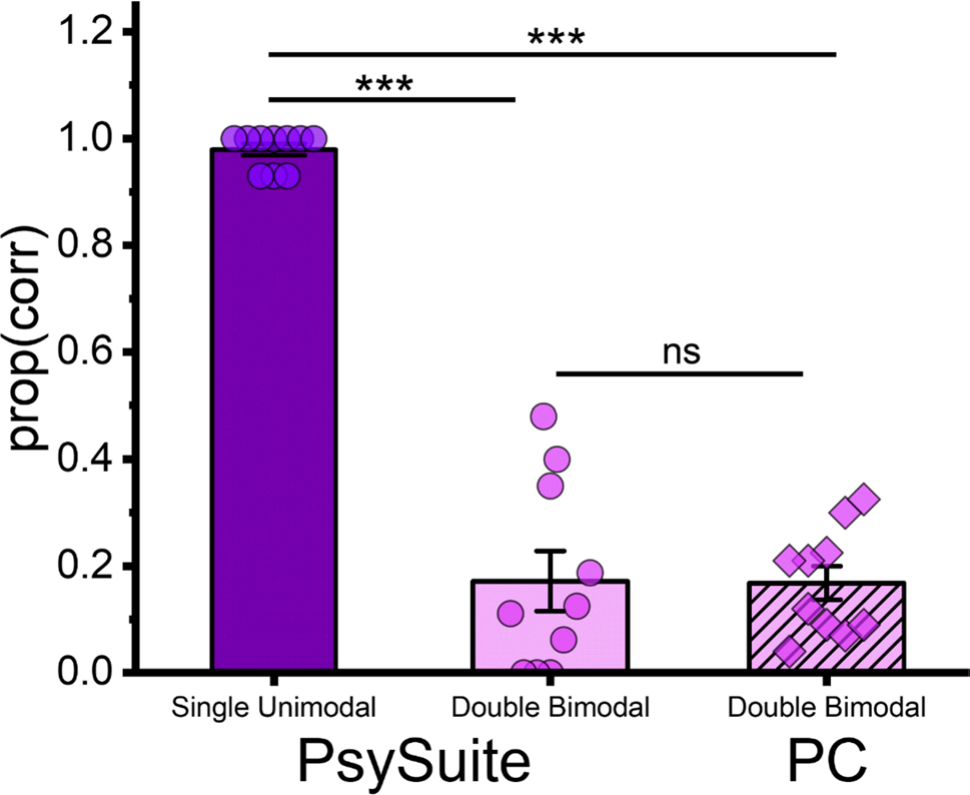
DFI elicited with *PsySuite* and a classic PC-based setup. Proportion of correct responses for the single uni-modal (one flash displayed) and the double bi-modal (one flash and two sounds delivered) conditions, tested using *PsySuite* and a classic PC-based setup. *Single points* indicate individual participants, while *error bars* indicate ± SEM (*** = *p* value < 0.001, ns = *p* value > 0.05)

To compare performances across tasks and setups, we evaluated the difference in the proportion of correct responses between the single uni-modal and double bi-modal conditions, with the latter being completed on both the smartphone and PC-based setup (Fig. 12). Since the parent distribution from which our sample dataset was extracted is plausibly non-normal (i.e., in the single unimodal condition we expect that most of the participants reach 100% due to a ceiling effect), we opted for using a non-parametric approach, involving the Kruskal–Wallis test given its lower sensitivity to non-normal populations distributions (Lantz, 2013). We included in our analysis the proportion of correct responses as the dependent variable and condition as the only factor (three levels: single, double bimodal on smartphone, double bimodal on PC-based setup). Overall, we found a significant main effect of the factor condition (H(2) = 19.709, *p* < 0.001). Then, we performed post hoc comparisons using paired Dunn tests, correcting the final result using Bonferroni (considering a family of three). Overall, we found that the proportion of correct responses in the single unimodal condition (M = 0.97, SD = 0.034) was significantly higher than the proportion of correct responses measured in the double bi-modal one performed on a smartphone (M = 0.172, SD = 0.178; *z* = – 3.966, *p* < 0.001), as well as the double bi-modal performed using the classic PC-based setup (M = 0.168, SD = 0.1; z = – 3.71 *p* < 0.001). Most importantly, no statistically significant difference emerged across the two setups when performing the double bi-modal task (*z* = 0.256, *p* = 1).

### Behavioral conclusions

Through the DFI, a classic illusion widely studied within psychophysical research, we validated the use of *PsySuite* considering behavioral performance measured with the app. If *PsySuite* was not suitable for sustaining heavily demanding testing procedures in terms of hardware and software requirements, eliciting the DFI with it should have been almost impossible. The illusion requires short and accurate presentation timing that inevitably stresses the app's components. Thereby, eliciting the DFI with the app proves that such visual illusion, one of the most timing-dependent ones, is inducible using devices not inherently designed for research, such as smartphones, but adequately programmed.

The results of the performed behavioral validation are twofold. On the one hand, we managed to induce the DFI in all participants using *PsySuite*'s testing procedures, as their proportions of correct responses significantly dropped when two auditory stimuli and one visual stimulus were concurrently delivered. On the other hand, DFI magnitudes elicited with the app were equal to DFI magnitudes elicited with the classic PC-based setup, as the reduced proportion of correct responses in the double bi-modal condition did not differ between setups.

Taken together, these findings support *PsySuite*'s feasibility in performing remote and reliable psychophysical testing within the temporal domain, using short-timed auditory and visual stimuli. While *PsySuite*'s efficiency in delivering highly accurate and precise stimulations was already supported by the hardware validation described in the previous section, behavioral validation confirmed that *PsySuite* is a powerful tool for collecting behavioral data. Notably, behavioral validation was developed to control the influence of factors potentially interfering with results collected when using smartphones to deliver psychophysical testing procedures. To name but a few examples, behavioral results could be influenced by the fact that data collection was performed in an environment known by the participant (e.g., their living room) or that the experimenter was not near the participant during the procedure.

Overall, *PsySuite* validations demonstrated that obtaining psychophysical measurements does not require participants to reach research facilities. Data collection can be safely and remotely performed at their own houses with just an everyday, easy-to-use device such as a smartphone.

## Discussion

In recent years, collecting sufficient and reliable data to support experimental hypotheses has been far from effortless. Considering the 2020 COVID-19 pandemic and the fact that testing hypotheses might require comparing performances between typical participants and populations of reference, which are challenging to recruit, research can sometimes significantly slow down. Regardless of recruiting issues that emerged during the pandemic, moving laboratory data collection procedures to a more user-friendly, ecological setting has always been an exciting possibility. We thereby designed, developed, and validated *PsySuite*; an Android app focused on promoting a remote, handy, and safe approach to psychophysical data collection. Indeed, *PsySuite* was created to allow participants to perform classic psychophysical experiments in a secure and ecological environment without necessarily reaching research facilities or universities. By granting Internet access to the smartphone, using a SIM card, or simply connecting it to an existing WI-FI™ network, results are directly sent to the experimenter, who can start analyzing the data right after the participant completes the task. Considering that, by 2021, the share of EU households with Internet access had risen to 92% and that broadband Internet access was used by 90% of the households in the EU in the same year, sending experiments' results using this procedure is highly efficient in most cases.

*PsySuite*'s reliability was assessed via both hardware and behavioral validation. Hardware validation was pursued to ensure that both stimulus durations and minimum temporal differences achievable between stimulations matched the input values chosen by the experimenter. For completion purposes, *PsySuite*'s stability was investigated using two different midrange smartphone models: a Xiaomi Mi A2, produced in 2018, and a Samsung A40, firstly released in 2019. Conversely, behavioral validation was conducted to test whether the double-flash illusion (DFI) was equally eligible using *PsySuite* and a classic PC-based setup. The DFI was chosen because it requires extremely short and precise presentation timing to be successfully elicited, significantly stressing the smartphone's hardware components.

The first important consideration is that the app managed to deliver both auditory and visual stimulations with satisfactory accuracy and precision in any possible combination, regardless of the smartphone model involved. However, tactile stimulations lacked similar faithfulness, being seriously unreliable when using the Samsung A40 model and reaching acceptable accuracy and precision only when using the Xiaomi smartphone. The second important consideration is that the software components significantly determined stimuli durations, especially for the visual stimuli (whose minimum duration was tied to the display's refresh rate). Auditory stimuli were delivered with extreme accuracy and precision in all considered cases. At the same time, the lack of control emerged when producing tactile stimulations with the Samsung A40, and the slightly lower precision observed when using the Xiaomi is unequivocally determined by the smartphone's capacity to turn on and off the engine vibrators with extremely reliable timing. As hardware components are likely to become better and better in the following years, *PsySuite*'s reliability is nonetheless destined to increase due to future technical improvements available within the smartphone market. Anyway, we would like to stress that if planning to involve multimodal stimulations and not use the Xiaomi MI A2, an oscilloscope is needed to test the synchrony between stimuli presented across all various modalities. However, if the experimental design involves only unimodal stimulations, and the hardware specific of the smartphone are not significantly worse than the Xiaomi Mi A2 ones, we argue that performance will be reliable as no downgrade is expected when using more modern devices.

Furthermore, the comparison between the DFI elicited using *PsySuite*, and the more classic PC-based setup highlighted that comparable results could be collected using both experimental settings. Behavioral validation was needed to corroborate that participants could still validly perform psychophysical tasks even when using a smartphone, a tool not inherently designed for data collection. Many possible confounds can negatively impact performance when using *PsySuite* in an unsupervised environment (e.g., at participants' homes), some of them being even extremely hard to define. We thereby decided to foster this comparison to ensure that data collected with this novel approach resembles similar scientific validity of data collected within the more controlled laboratory environment. For example, participants' commitment could have varied due to their familiarity with the surroundings, or using a smartphone instead of a PC might have altered the task's engagement. Since not only the DFI was induced using *PsySuite*, but also the illusion's magnitude was comparable to the one elicited using a classic PC-based setup held in a research facility, the app proved itself to be once more a reliable tool to perform psychophysical testing. These findings utterly support *PsySuite*'s feasibility in promoting a remote approach to data collection and suggest that behavioral measurements obtained with the app are a reliable approximation of human performance, even though they were collected with such an unorthodox and novel approach.

One of the issues that often arise when performing remote data collection with already available tools, i.e., using online platforms, is that it is hard to balance the performances of each participant's PC. This is especially problematic when using stimuli that must be temporally accurate and whose duration must be stable across multiple trials. Whether not properly matched, less performing hardware components might lead to a misrepresentation of the stimuli during the procedure, thus biasing collected results. With *PsySuite,* the chance of this occurrence has been virtually reduced to zero since all users can use exactly the same hardware: Moreover, hardware requirements are not so demanding, as proven by the fact that even a mid-range smartphone produced in 2018 (the Xiaomi Mi A2) demonstrated to be a solid device through which develop psychophysical testing and are expected to increase year by year. However, we would like to stress again that researchers willing to use other models need to validate their performance with an oscilloscope, and insert into the app the between-modalities delays, which is the only values that are expected to change on a model-by-model basis.

Given the recent pandemic outbreak, the benefits of a remote and safe data collection approach are more than evident. Nonetheless, the novel approach to psychophysical testing that we are proposing is favorable for more than the ability to tackle social distancing limitations that have slowed down research since March 2020. First, *PsySuite*'s dissemination will significantly boost large-scale data collections while maintaining the constancy of setups between participants, an aspect that is hardly controllable with online platforms. Second, *PsySuite*'s portability will be a great addition when designing psychophysical data collection in specific populations with mobility issues, such as hospitalized patients and paraplegic individuals, to name a few. Third, such portability will also facilitate the implementation of time-consuming experimental design, such as perceptual learning protocols, which require several days of training to be effective. In fact, using *PsySuite,* participants can perform training sessions at their homes, increasing the users' comfort and reducing economic expenses if the institution pays participants to come to the facility. Lastly, with *PsySuite,* it will be possible to develop specific user-centered testing procedures, such as successfully reducing the test anxiety that sometimes distress (especially, but not uniquely) the youngest participants who perform experiments in an unknown environment. Allowing psychophysical procedures to be performed in a familiar setting (e.g., their homes or a rehabilitation center attended long-time) will indeed be perceived as a less stressful experience, therefore supporting data collection on participants that otherwise would have probably not completed the task.

## Supplementary Information

Below is the link to the electronic supplementary material.

Supplementary file1 (PDF 2770 kb)

## Data Availability

All data used in this study can be accessed at the following link: https://zenodo.org/records/10629206.

## Code availability

PsySuite, its source code, and all related documentation is freely available at the following link: https://gitlab.iit.it/u-vip_public/psysuite/app.

## References

[CR1] Anobile, G., Arrighi, R., Togoli, I., & Burr, D. C. (2016). A shared numerical representation for action and perception. *eLife*, *5*, e16161. 10.7554/eLife.16161 10.7554/eLife.16161 PMC4978523 27504969

[CR2] Anobile, G., Domenici, N., Togoli, I., Burr, D., & Arrighi, R. (2020). Distortions of visual time induced by motor adaptation. *Journal of Experimental Psychology: General*, *149*(7), Article 7. 10.1037/xge0000709 31789572

[CR3] Anwyl-Irvine, A., Dalmaijer, E. S., Hodges, N., & Evershed, J. K. (2021). Realistic precision and accuracy of online experiment platforms, web browsers, and devices. *Behavior Research Methods,**53*(4), 1407–1425. 10.3758/s13428-020-01501-5 33140376 10.3758/s13428-020-01501-5 PMC8367876

[CR4] Anwyl-Irvine, A. L., Massonnié, J., Flitton, A., Kirkham, N., & Evershed, J. K. (2020). Gorilla in our midst: An online behavioral experiment builder. *Behavior Research Methods,**52*(1), 388–407. 10.3758/s13428-019-01237-x 31016684 10.3758/s13428-019-01237-x PMC7005094

[CR5] Bhavnani, S., Mukherjee, D., Bhopal, S., Sharma, K. K., Dasgupta, J., Divan, G., Soremekun, S., Roy, R., Kirkwood, B., & Patel, V. (2021). The association of a novel digital tool for assessment of early childhood cognitive development, ‘DEvelopmental assessment on an E-Platform (DEEP)’, with growth in rural India: A proof of concept study. *eClinicalMedicine*, *37*. 10.1016/j.eclinm.2021.100964 10.1016/j.eclinm.2021.100964 PMC8225699 34195580

[CR6] Bignardi, G., Dalmaijer, E. S., Anwyl-Irvine, A., & Astle, D. E. (2021). Collecting big data with small screens: Group tests of children’s cognition with touchscreen tablets are reliable and valid. *Behavior Research Methods,**53*(4), 1515–1529. 10.3758/s13428-020-01503-3 33269446 10.3758/s13428-020-01503-3 PMC7710155

[CR7] Binda, P., Cicchini, G. M., Burr, D. C., & Morrone, M. C. (2009). Spatiotemporal distortions of visual perception at the time of saccades. *Journal of Neuroscience*, *29*(42), Article 42. 10.1523/JNEUROSCI.3723-09.2009 10.1523/JNEUROSCI.3723-09.2009 PMC6665185 19846702

[CR8] Domenici, N., Inuggi, A., Tonelli, A., & Gori, M. (2021). A novel Android app to evaluate and enhance auditory and tactile temporal thresholds. *2021 43rd Annual International Conference of the IEEE Engineering in Medicine & Biology Society (EMBC)*, 5885–5888. 10.1109/EMBC46164.2021.9630028 10.1109/EMBC46164.2021.9630028 34892458

[CR9] Domenici, N., Tonelli, A., & Gori, M. (2021b). Adaptation to high-frequency vibrotactile stimulations fails to affect the clock in young children. *Current Research in Behavioral Sciences,**2*, 100018. 10.1016/j.crbeha.2021.100018

[CR10] Engel, A. K., & Singer, W. (2001). Temporal binding and the neural correlates of sensory awareness. *Trends in Cognitive Sciences,**5*(1), 16–25. 10.1016/S1364-6613(00)01568-0 11164732 10.1016/s1364-6613(00)01568-0

[CR11] Fechner, G. T. (1948). Elements of psychophysics, 1860. In *Readings in the history of psychology* (pp. 206–213). Appleton-Century-Crofts. 10.1037/11304-026

[CR12] Finger, H., Goeke, C., Diekamp, D., Standvoss, K., & König, P. (2017). *LabVanced: A Unified JavaScript Framework for Online Studies*.

[CR13] Fornaciai, M., Arrighi, R., & Burr, D. C. (2016). Adaptation-induced compression of event time occurs only for translational motion. *Scientific Reports,**6*(1), 23341. 10.1038/srep23341 27003445 10.1038/srep23341 PMC4802346

[CR14] Giamattei, M., Yahosseini, K. S., Gächter, S., & Molleman, L. (2020). LIONESS Lab: A free web-based platform for conducting interactive experiments online. *Journal of the Economic Science Association,**6*(1), 95–111. 10.1007/s40881-020-00087-0

[CR15] Gokul, G., Yan, Y., Dantu, K., Ko, S. Y., & Ziarek, L. (2016). Real-Time Sound Processing on Android. *Proceedings of the 14th International Workshop on Java Technologies for Real-Time and Embedded Systems*, 1–10. 10.1145/2990509.2990512

[CR16] Gori, M., Bollini, A., Maviglia, A., Amadeo, M. B., Tonelli, A., Crepaldi, M., & Campus, C. (2019). MSI Caterpillar: An Effective Multisensory System to Evaluate Spatial Body Representation. *IEEE International Symposium on Medical Measurements and Applications (MeMeA),**2019*, 1–6. 10.1109/MeMeA.2019.8802133

[CR17] Gori, M., Sandini, G., & Burr, D. (2012). Development of visuo-auditory integration in space and time. *Frontiers in Integrative Neuroscience*, *6*. https://www.frontiersin.org/articles/10.3389/fnint.2012.00077 10.3389/fnint.2012.00077 PMC3443931 23060759

[CR18] Hirst, R. J., McGovern, D. P., Setti, A., Shams, L., & Newell, F. N. (2020). What you see is what you hear: Twenty years of research using the sound-induced flash illusion. *Neuroscience & Biobehavioral Reviews,**118*, 759–774. 10.1016/j.neubiorev.2020.09.006 32937116 10.1016/j.neubiorev.2020.09.006

[CR19] Inuggi, A., Tonelli, A., & Gori, M. (2021). PsySuite, an Android app for behavioural tests in the temporal domain. *IEEE International Symposium on Medical Measurements and Applications (MeMeA),**2021*, 1–6. 10.1109/MeMeA52024.2021.9478724

[CR20] Ioannidis, J. P. A. (2008). Why most discovered true associations are inflated. *Epidemiology (Cambridge, Mass.)*, *19*(5), 640–648. 10.1097/EDE.0b013e31818131e7 10.1097/EDE.0b013e31818131e7 18633328

[CR21] Keil, J. (2020). Double-flash illusions: Current findings and future directions. *Frontiers in Neuroscience*, *14*. https://www.frontiersin.org/journals/neuroscience/articles/10.3389/fnins.2020.00298 10.3389/fnins.2020.00298 PMC7146460 32317920

[CR22] Lange, J., Oostenveld, R., & Fries, P. (2011). Perception of the touch-induced visual double-flash illusion correlates with changes of rhythmic neuronal activity in human visual and somatosensory areas. *NeuroImage,**54*(2), 1395–1405. 10.1016/j.neuroimage.2010.09.031 20854915 10.1016/j.neuroimage.2010.09.031

[CR23] Lantz, B. (2013). The impact of sample non-normality on ANOVA and alternative methods. *British Journal of Mathematical and Statistical Psychology,**66*(2), 224–244. 10.1111/j.2044-8317.2012.02047.x 22624658 10.1111/j.2044-8317.2012.02047.x

[CR24] Lukács, G., & Gartus, A. (2023). Precise display time measurement in JavaScript for web-based experiments. *Behavior Research Methods,**55*(3), 1079–1093. 10.3758/s13428-022-01835-2 35581437 10.3758/s13428-022-01835-2

[CR25] Marin-Campos, R., Dalmau, J., Compte, A., & Linares, D. (2021). StimuliApp: Psychophysical tests on mobile devices. *Behavior Research Methods,**53*(3), 1301–1307. 10.3758/s13428-020-01491-4 33037602 10.3758/s13428-020-01491-4 PMC8219581

[CR26] McCormick, D., & Mamassian, P. (2008). What does the illusory-flash look like? *Vision Research,**48*(1), 63–69. 10.1016/j.visres.2007.10.010 18054372 10.1016/j.visres.2007.10.010

[CR27] Murayama, K., Pekrun, R., & Fiedler, K. (2014). Research practices that can prevent an inflation of false-positive rates. *Personality and Social Psychology Review,**18*(2), 107–118. 10.1177/1088868313496330 23965303 10.1177/1088868313496330

[CR28] Pitchford, N. J., & Outhwaite, L. A. (2016). Can touch screen tablets be used to assess cognitive and motor skills in early years primary school children? A cross-cultural study. *Frontiers in Psychology*, *7*. https://www.frontiersin.org/journals/psychology/articles/10.3389/fpsyg.2016.01666 10.3389/fpsyg.2016.01666 PMC5078468 27826281

[CR29] Pronk, T., Wiers, R. W., Molenkamp, B., & Murre, J. (2020). Mental chronometry in the pocket? Timing accuracy of web applications on touchscreen and keyboard devices. *Behavior Research Methods,**52*(3), 1371–1382. 10.3758/s13428-019-01321-2 31823223 10.3758/s13428-019-01321-2 PMC7280355

[CR30] Rammsayer, T. H., & Lima, S. D. (1991). Duration discrimination of filled and empty auditory intervals: Cognitive and perceptual factors. *Perception & Psychophysics,**50*(6), 565–574. 10.3758/BF03207541 1780204 10.3758/bf03207541

[CR31] Robinson, S. J., & Brewer, G. (2016). Performance on the traditional and the touch screen, tablet versions of the Corsi Block and the Tower of Hanoi tasks. *Computers in Human Behavior,**60*, 29–34. 10.1016/j.chb.2016.02.047

[CR32] Semmelmann, K., Nordt, M., Sommer, K., Röhnke, R., Mount, L., Prüfer, H., Terwiel, S., Meissner, T. W., Koldewyn, K., & Weigelt, S. (2016). U can touch this: How tablets can be used to study cognitive development. *Frontiers in Psychology*, *7*. https://www.frontiersin.org/journals/psychology/articles/10.3389/fpsyg.2016.01021 10.3389/fpsyg.2016.01021 PMC4935681 27458414

[CR33] Shams, L., Kamitani, Y., & Shimojo, S. (2000). What you see is what you hear. *Nature,**408*(6814), 788–788. 10.1038/35048669 11130706 10.1038/35048669

[CR34] Simmons, J. P., Nelson, L. D., & Simonsohn, U. (2011). False-positive psychology: Undisclosed flexibility in data collection and analysis allows presenting anything as significant. *Psychological Science,**22*(11), 1359–1366. 10.1177/0956797611417632 22006061 10.1177/0956797611417632

[CR35] Stoet, G. (2017). PsyToolkit: A novel web-based method for running online questionnaires and reaction-time experiments. *Teaching of Psychology,**44*(1), 24–31. 10.1177/0098628316677643

[CR36] Togoli, I., Marlair, C., Collignon, O., Arrighi, R., & Crollen, V. (2021). Tactile numerosity is coded in external space. *Cortex,**134*, 43–51. 10.1016/j.cortex.2020.10.008 33249299 10.1016/j.cortex.2020.10.008

[CR37] Tonelli, A., Cuturi, L. F., & Gori, M. (2017). The influence of auditory information on visual size adaptation. *Frontiers in Neuroscience*, *11*. https://www.frontiersin.org/journals/neuroscience/articles/10.3389/fnins.2017.00594 10.3389/fnins.2017.00594 PMC5660698 29114201

[CR38] Tonelli, A., Pooresmaeili, A., & Arrighi, R. (2020). The role of temporal and spatial attention in size adaptation. *Frontiers in Neuroscience*, *14*. https://www.frontiersin.org/journals/neuroscience/articles/10.3389/fnins.2020.00539 10.3389/fnins.2020.00539 PMC7279953 32514266

[CR39] Violentyev, A., Shimojo, S., & Shams, L. (2005). Touch-induced visual illusion. *NeuroReport,**16*(10), 1107–1110. 10.1097/00001756-200507130-00015 15973157 10.1097/00001756-200507130-00015

