# Supplementary material for: PsySuite: An android application designed to perform multimodal psychophysical testing
Source: Behav Res Methods. 2024 Aug 13;56(8):8308–29. doi: 10.3758/s13428-024-02475-4 (PMC11525261; doi:10.3758/s13428-024-02475-4)
Supplement: Supplementary file 1 — Supplementary file1 (PDF 2770 kb) [file 13428_2024_2475_MOESM1_ESM.pdf]

Supplementary Information

PsySuite: an Android Application designed to perform multimodal psychophysical testing

Alberto Inuggi, Nicola Domenici, Alessia Tonelli, & Monica Gori

Test creation Tutorial

This tutorial will guide users to create a simple reaction time (RT) experiment composed of three sub-task, one for each available sensory modality. The app will present the stimulus in the selected modality and the user shall press a button as fast as possible. The App will record the time elapsed between stimulus delivery and subject button press in a proper text file.

1) Create a package in iit.psysuite.core.tests

2) create there a python class: TestRT

make it derive from TestBasic and add the following parameters in the constructor

```
class TestRT(ctx: Context, activity: Activity, hostfragment: Fragment, subject:
SubjectBasicParcel, vibrator: VibrationManager?, mImageView: ImageView?,
speechManager:SpeechManager?) : TestBasic(ctx, activity, hostfragment, subject,
vibrator, mImageView, speechManager)
```

The editor warns you that four abstract methods of TestBasic need to be defined.

- InitTest
- onTrialEnd
- show
- initSummary

Let the editor do it for you.

3) In the companion object of iit.psysuite.core.test.TestBasic, define a unique code for each of the sub-task

```
@JvmStatic val TEST_RT_AUDIO = 1
@JvmStatic val TEST_RT_TACTILE = 2
@JvmStatic val TEST_RT_VISUAL = 3
```

One for each sensorial modality.

4) In iit.psysuite.core.ui.fragments.TestFragment.onActivityCreated add the following code in the branch of the command:

```
when(mSubjectParcel!!.type){
```

```
TestBasic.TEST_RT_AUDIO,
TestBasic.TEST_RT_TACTILE,
TestBasic.TEST_RT_VISUAL -> mTest = TestRT(requireContext(),
requireActivity(), this, mSubjectParcel!!, vibrator, binding.circleView,
speechManager)
```

5) Define the following properties of interest:

create the LOG_TAG for logging functions and create the companion object to define static properties

```
override var LOG_TAG:String = TestRT::class.java.simpleName
companion object {
   @JvmStatic val TEST_BASIC_LABEL                  = "RT"   // will be
written in result files
   @JvmStatic var NUM_TRIALS                        = 32
   @JvmStatic val STIMULUS_DURATION_VISUAL:Long     = 50
   @JvmStatic val STIMULUS_DURATION_TACTILE:Long    = 50
   @JvmStatic val STIMULUS_DURATION_AUDIO:Long      = 50

   @JvmStatic val STIMULUS_TYPE_AUDIO               = "AUDIO"
   @JvmStatic val STIMULUS_TYPE_TACTILE             = "TACTILE"
   @JvmStatic val STIMULUS_TYPE_VISUAL              = "VISUAL"
   @JvmStatic val STIMULUS_TYPE_AUDIO_LOG           = "A"
   @JvmStatic val STIMULUS_TYPE_TACTILE_LOG         = "T"
   @JvmStatic val STIMULUS_TYPE_VISUAL_LOG          = "V"
```

then add two static methods

```
   fun getConditionsInfo(ctx: Context): List<ConditionData>{
       return if(VibrationManager.sysHasVibrator(ctx))
           mutableListOf(
           ConditionData(TEST_BASIC_LABEL + "_" +
STIMULUS_TYPE_AUDIO           , TEST_RT_AUDIO          ,
"${TEST_BASIC_LABEL}${STIMULUS_TYPE_AUDIO_LOG}"          ,
Populations.hearing_populations),
           ConditionData(TEST_BASIC_LABEL + "_" +
STIMULUS_TYPE_TACTILE         , TEST_RT_TACTILE        ,
"${TEST_BASIC_LABEL}${STIMULUS_TYPE_TACTILE_LOG}"        ,
Populations.all_populations),
           ConditionData(TEST_BASIC_LABEL + "_" +
STIMULUS_TYPE_VISUAL          , TEST_RT_VISUAL         ,
"${TEST_BASIC_LABEL}${STIMULUS_TYPE_VISUAL_LOG}"         ,
Populations.sighted_populations),
       )
       else
           mutableListOf(
               ConditionData(TEST_BASIC_LABEL + "_" +
STIMULUS_TYPE_AUDIO           , TEST_RT_AUDIO          ,
"${TEST_BASIC_LABEL}${STIMULUS_TYPE_AUDIO_LOG}"          ,
Populations.hearing_populations),
               ConditionData(TEST_BASIC_LABEL + "_" +
STIMULUS_TYPE_VISUAL          , TEST_RT_VISUAL         ,
```

```
"${TEST_BASIC_LABEL}${STIMULUS_TYPE_VISUAL_LOG}"          ,
Populations.sighted_populations),
           )
   }

   fun getNextTrialModes(ctx: Context):List<List<Int>>{
       return if(VibrationManager.sysHasVibrator(ctx))
           listOf( listOf(TEST_NEXTTRIAL_NOCHOOSE),
listOf(TEST_NEXTTRIAL_NOCHOOSE), listOf(TEST_NEXTTRIAL_NOCHOOSE))
       else
           listOf( listOf(TEST_NEXTTRIAL_NOCHOOSE),
listOf(TEST_NEXTTRIAL_NOCHOOSE))
   }
}
```

getConditionsInfo is the way each test class exposes the available sub-tasks to the SubjectDialogFragment. Each element of the list correspond to an entry of its combobox Conditions;

getNextTrialModes defines what should happen at the end of a trial. In this case the TEST_NEXTTRIAL_NOCHOOSE flag says that the subject does not have to do anything and the next trial can start.

Both methods accept a single parameter of type Context that has two main functions, access resources (e.g. text to display) and verify whether the device can vibrate or not (calling VibrationManager.sysHasVibrator(ctx)).

In the latter case, sub-tasks involving tactile stimuli are not displayed in the User info dialog and thus cannot be selected.

Other options are:

```
@JvmStatic val TEST_NEXTTRIAL_AUTO = 0 // user can select to go directly to
next trial or abort
@JvmStatic val TEST_NEXTTRIAL_BUTTON = 1 // user can select to wait and then
press a NEXT button
@JvmStatic val TEST_NEXTTRIAL_ANSWER = 2 // wait for ANSWER dialog
@JvmStatic val TEST_NEXTTRIAL_VOICE_ANSWER = 3 // wait for VOICE ANSWER
dialog through speech recognition
@JvmStatic val TEST_NEXTTRIAL_VOICE_NORMAL_ANSWER = 4 // wait for either
ANSWER dialog or VOICE ANSWER through speech recognition
```

Define how to present each modality. Audio stimuli can be created from four different sources (tone, high latency wav, low latency with AudioTrack and very low latency with Oboe. Visual stimuli can be presented in two different ways, making them visible or invisible or make them switch from one image (off state) and another one (on state). Tactile stimuli can be single stimuli or predetermined sequences.

Visual and tactile stimuli performance differences were not investigated, and the best option (StimuliManager.STIM_TYPE_V1 or STIM_TYPE_V2, StimuliManager.STIM_TYPE_T1 or STIM_TYPE_T2) should be selected according to the requested scenario. Audio stimuli for psychophysics tasks must use the Oboe method (StimuliManager.STIM_TYPE_A4). In case of several long audio files that do not need very fast performance can be played back with Media player API (StimuliManager.STIM_TYPE_A2).

```
private var STIM_A = StimuliManager.STIM_TYPE_A4
private var STIM_V = StimuliManager.STIM_TYPE_V2
private var STIM_T = StimuliManager.STIM_TYPE_T1
```

Visual stimuli must be defined:

```
override var mDrawablesResource: MutableList<Int> = mutableListOf(R.drawable.white_circle,
R.drawable.blue_circle)
```

In case of white background the off state could the first element of such list

6) init and set up task data. Write initTest.

This method is automatically called by the TestFragment method. User must implement there the following steps:

- Sanity checks
- Define stimuli characteristics
- Create task trials
- Send EVENT_TEST_SETUP_COMPLETED event

If the task can use tactile stimulation, verify that the vibrator property is not null. This variable is automatically calculated by the App at its startup. For example, tablets usually do not have vibrator engines and this variable is null.

```
override fun initTest(){
when {
mImageView == null -> throw
ImageViewDefinedException("IMAGE_VIEW_NOT_DEFINED")
vibrator == null -> throw VibratorNotDefinedException("VIBRATOR_NOT_DEFINED")
}
```

Set the task label according to subject.type

```
mTestLabel = ""
getConditionsInfo(ctx).map {
    if (it.id == subject.type) mTestLabel = it.label
}
if(mTestLabel.isEmpty()) throw Exception("ERROR in TestRT.initTest: type code
was not recognized")
```

Since vibrator engines also produce a tiny sound, it is advisable to add a white noise to all those tasks involving multimodal stimulation. Users asked to attend tactile stimuli could instead implicitly attend its sound

```
mNoise = AudioManager.getAudioResource(ctx,"wnoise_20s", 0.01f)
```

If this line is omitted (or contained within an if) the mNoise property remains null and further calls like mNoise?.start() do not have any effect

Define the source of each stimulus.

```
mStimuliManager = StimuliManager(
    AudioManager(STIM_A, audioResources[STIMULUS_DURATION_AUDIO] ?:
"t1000hz_50ms.wav", duration = STIMULUS_DURATION_AUDIO, ctx = ctx, handler =
mStimuliHandler),
    TactileManager(vibrator!!, duration = STIMULUS_DURATION_TACTILE, handler =
mStimuliHandler, type = STIM_T),
    VisualManager(STIM_V, mImageView!!, mDrawablesResource[1],
mDrawablesResource[0], duration = STIMULUS_DURATION_VISUAL, handler =
mStimuliHandler),
    delaysAligner, ctx, mStimuliHandler)
```

Now it's time to create the task trials

```
createTrials()
```

To define trials, the *mTrails* property of TestBasic must be filled with instances of TrialBasic or any of its subclasses.
The simplest trial, an instance of TrialBasic, has the following properties:

```
open class TrialBasic(var id:Int=-1, val type:Int, protected val
label:String="", var correct_answer:String="") {
```

A progressive id, a type code, a label and which is the correct answer (when applicable). In case of a RT task, all trials are of the same type and there isn't any correct or wrong answer.
The method createTrials, thus simply appends the mTrials list with instances of TrailBasic where only the id parameter differs, both type and label only reflect the sensory modality used.

```
private fun createTrials(){
    for(i in 0 until NUM_TRIALS)
        mTrials.add(TrialBasic(i, subject.type, mTestLabel))
}
```

At the end of *initTest*, when all set up is over, the test class can send the EVENT_TEST_SETUP_COMPLETED and the task can start

```
testEvent.accept(Pair(EVENT_TEST_SETUP_COMPLETED, null))
```

7) Show stimuli

Once TestFragment receives the event EVENT_TEST_SETUP_COMPLETED sent by TestRT instance, it does some internal stuff and then start the task, calling its show() method

```
override fun show(trial: TrialBasic, isRepeat: Boolean) {

   val stimulus_onset = (500L + random()*1000).toLong()
   mNoise?.start()
   mStimuliHandler.postDelayed({
```

```
        deliverStimulus(trial)
        testEvent.accept(Pair(EVENT_STIMULI_START, null))
    }, stimulus_onset)
}
```

Noise is started (if you defined it)
To reduce subjects expectation, define a random interval (500 < t < 1500 ms) before presenting the stimulus,

Signals' Sample Traces

As stated in the main text, for each possible combination of stimuli and sensory modalities we collected 100 simulations. Here, we reported all different signal sample traces, for both the Xiaomi MI A2 and the Samsung A40 smartphones. In all figures, green traces represent auditory signals, blue traces represent visual signals, and red traces represent tactile signals. Signals were rescaled when needed to increase readability.

Unimodal Single Stimulations (referring to Figure 6 in the main text)

Expected durations are, from left to right according to row placement, 7, 17, 30, 50, and 100ms respectively.

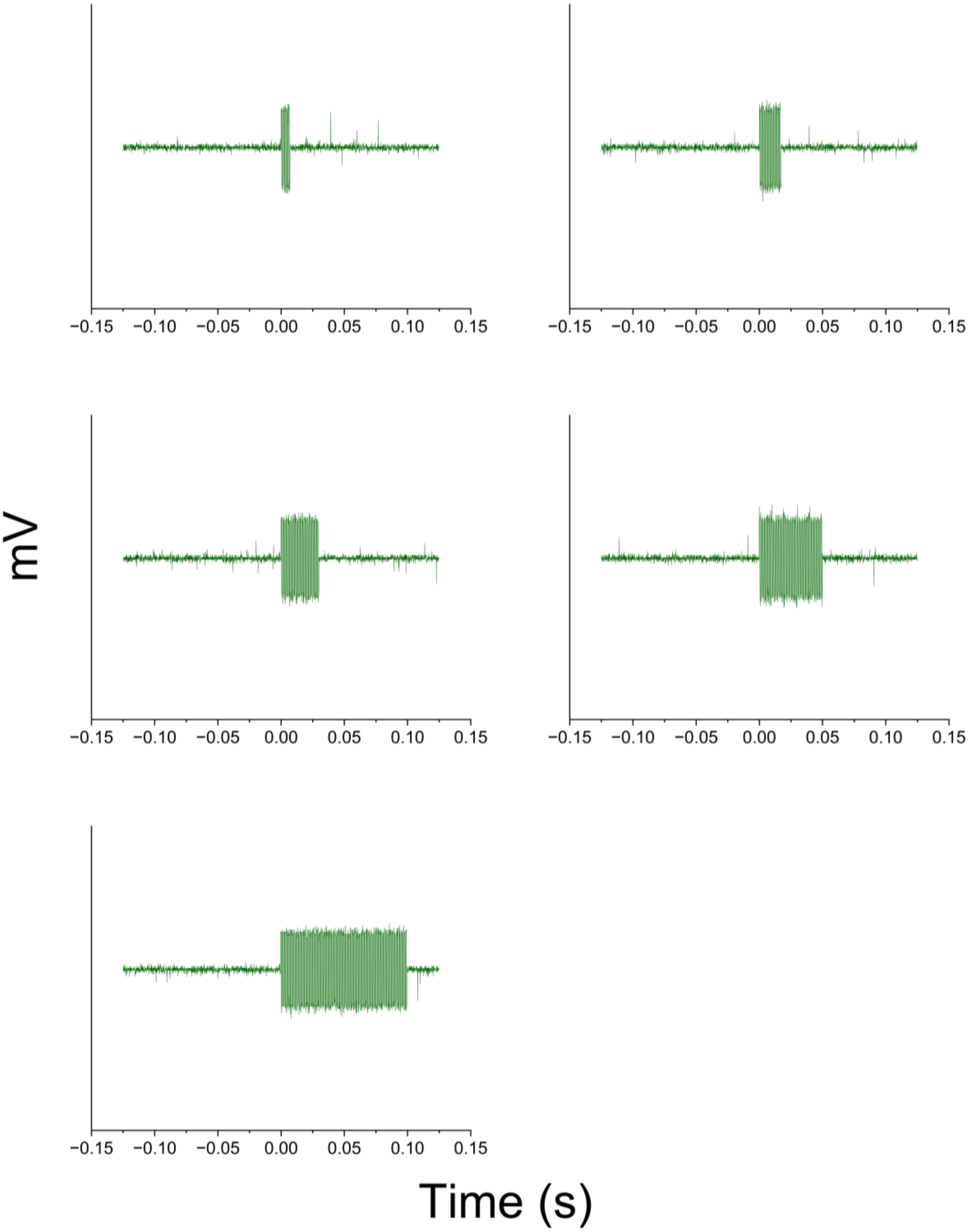

Figure 1S. Auditory single stimulations delivered by the Xiaomi MI A2 smartphone.

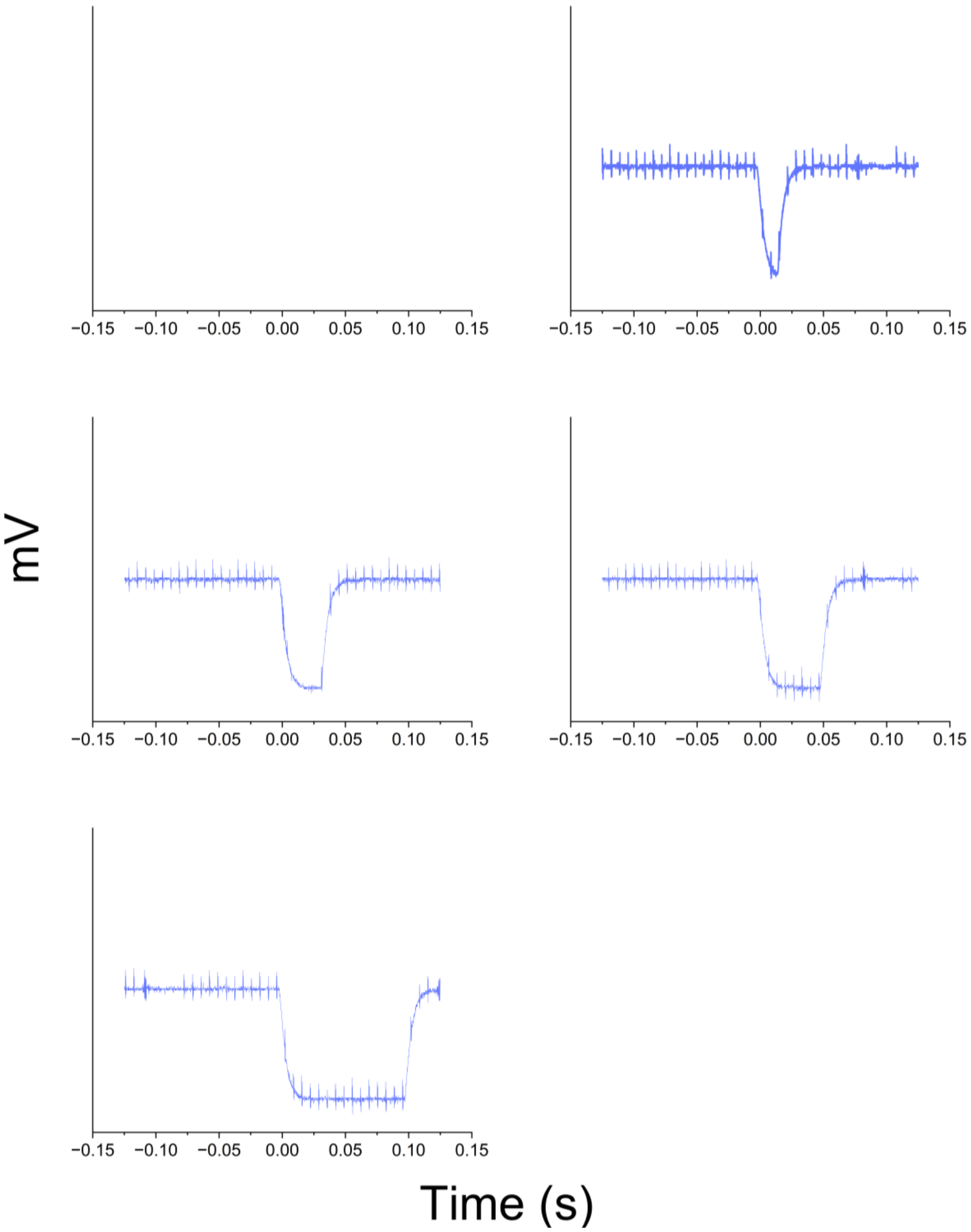

Figure 2S. Visual single stimulations delivered by the Xiaomi MI A2 smartphone.

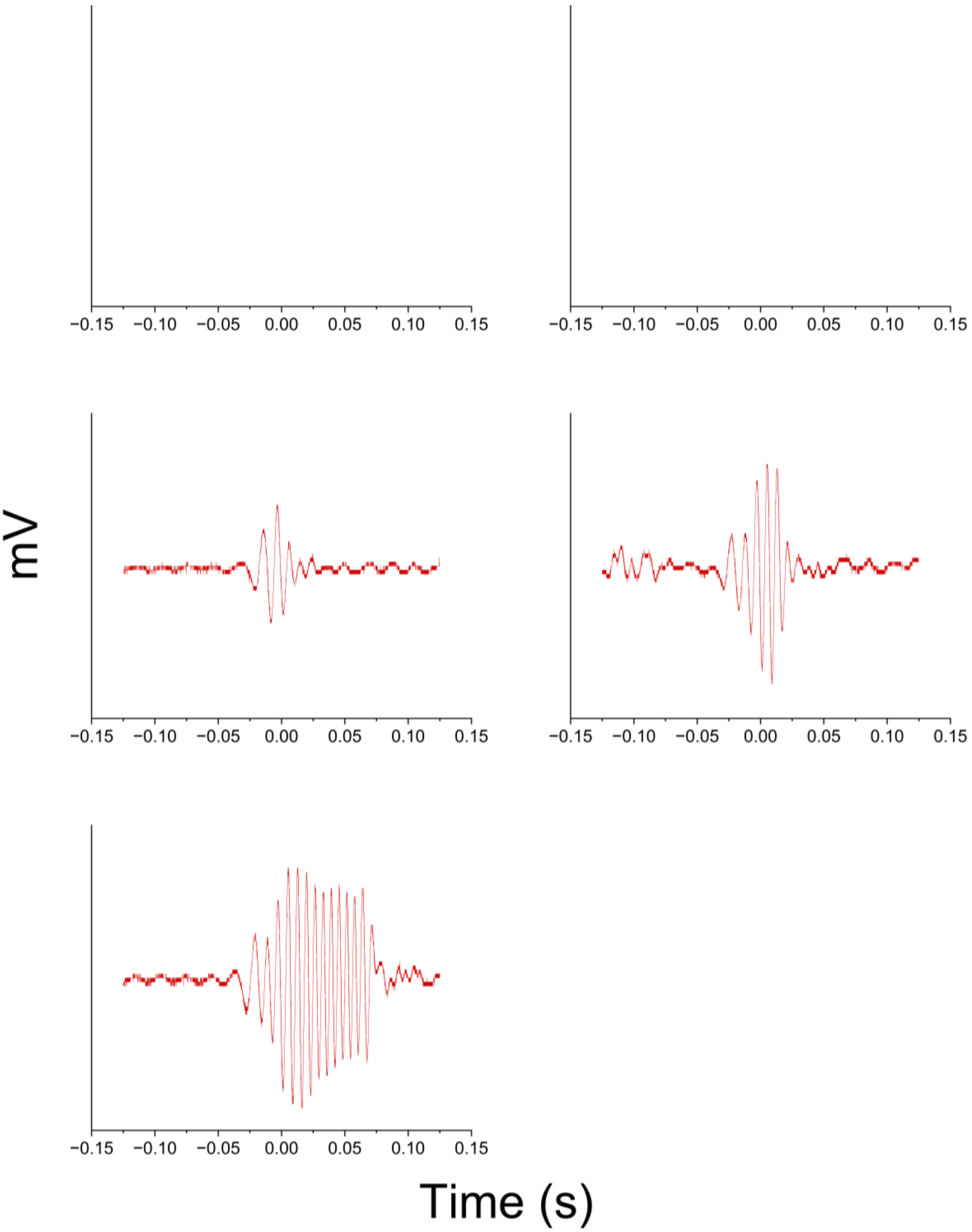

Figure 3S. Tactile single stimulations delivered by the Xiaomi MI A2 smartphone.

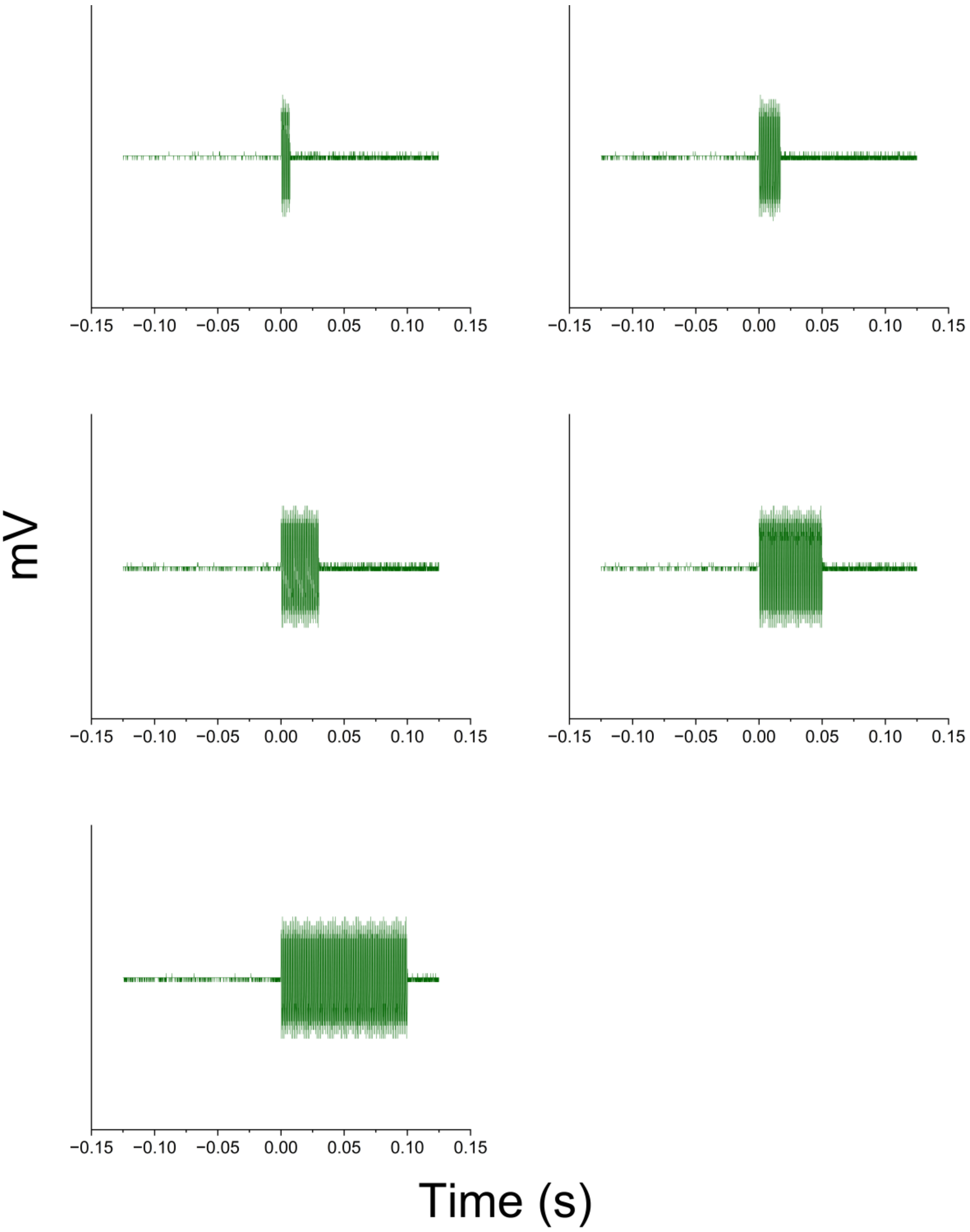

Figure 4S. Auditory single stimulations delivered by the Samsung A40 smartphone.

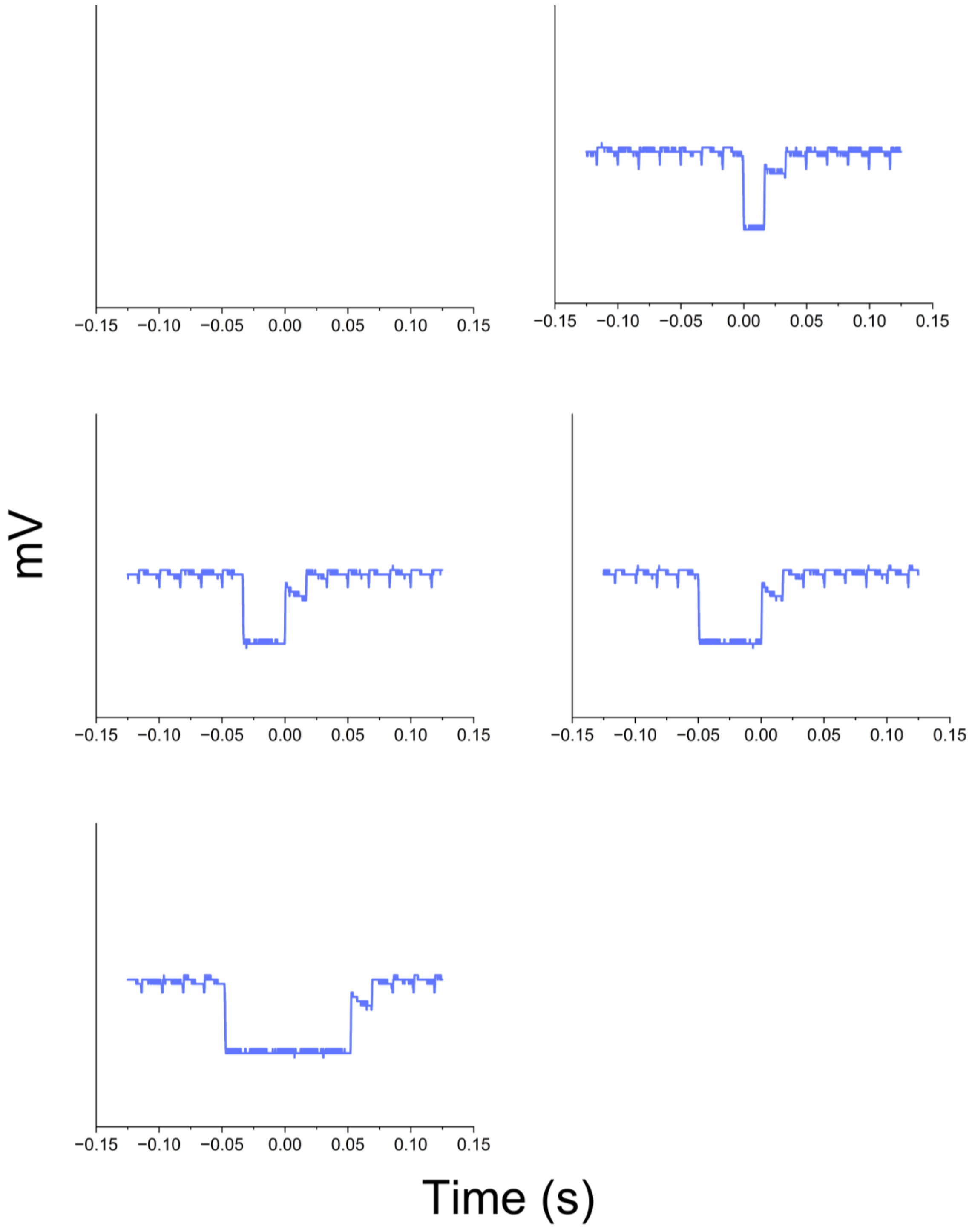

Figure 5S. Visual single stimulations delivered by the Samsung A40 smartphone.

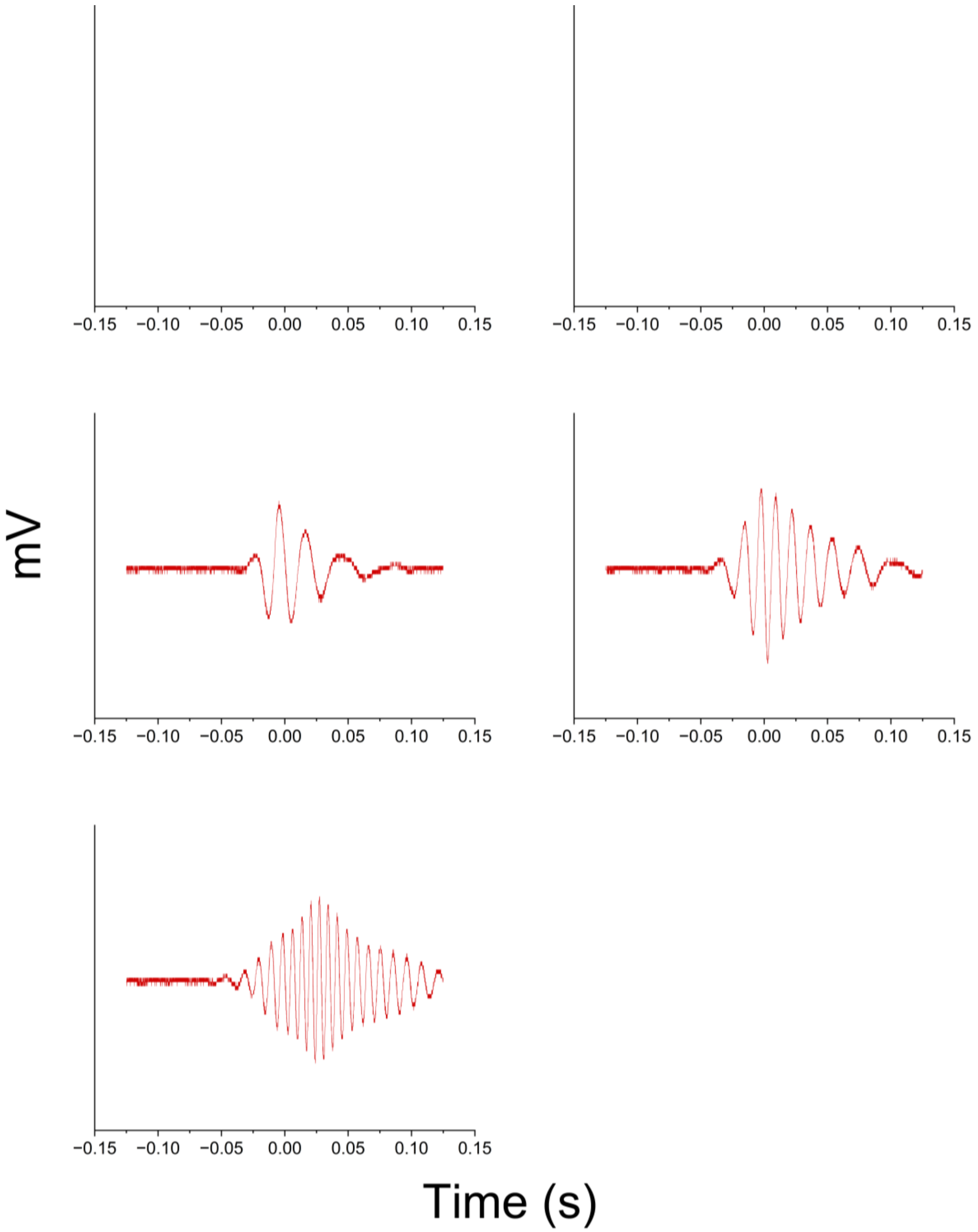

Figure 6S. Tactile single stimulations delivered by the Samsung A40 smartphone.

Unimodal Sequential Stimulations (referring to Figure 7 in the main text)

Expected durations-interval pairs are, from left to right according to row placement, 7-14, 17-34, 30-60, and 50-100ms, respectively.

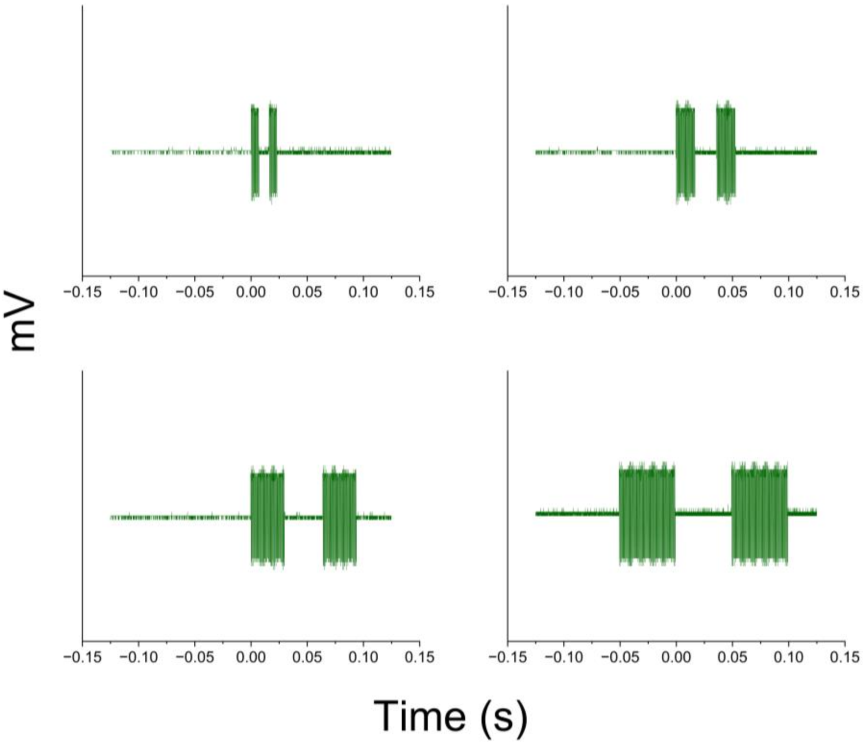

Figure 7S. Auditory sequential stimulations delivered by the Xiaomi MI A2 smartphone.

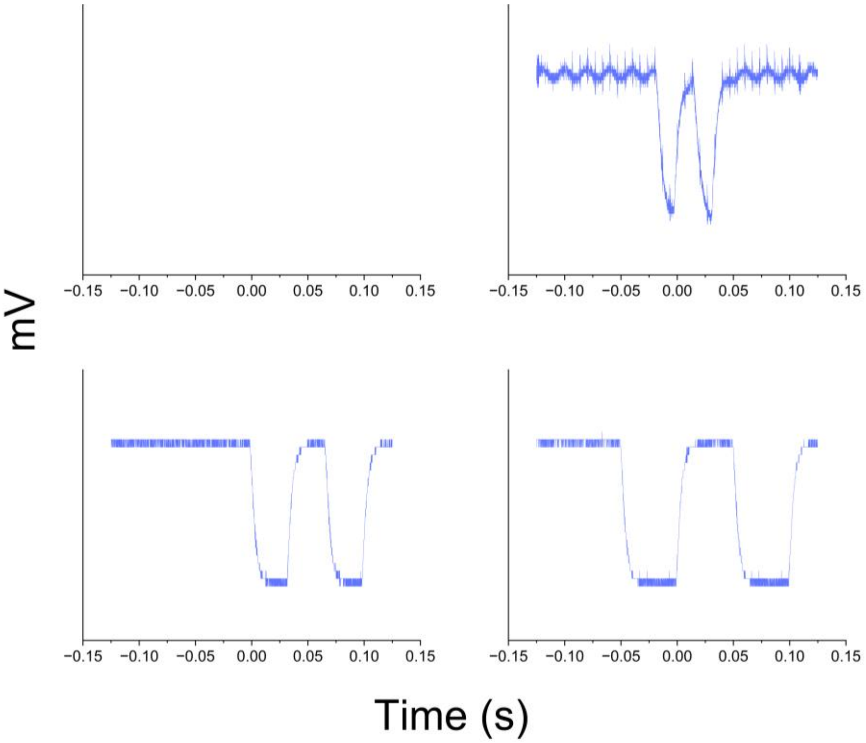

Figure 8S. Visual sequential stimulations delivered by the Xiaomi MI A2 smartphone.

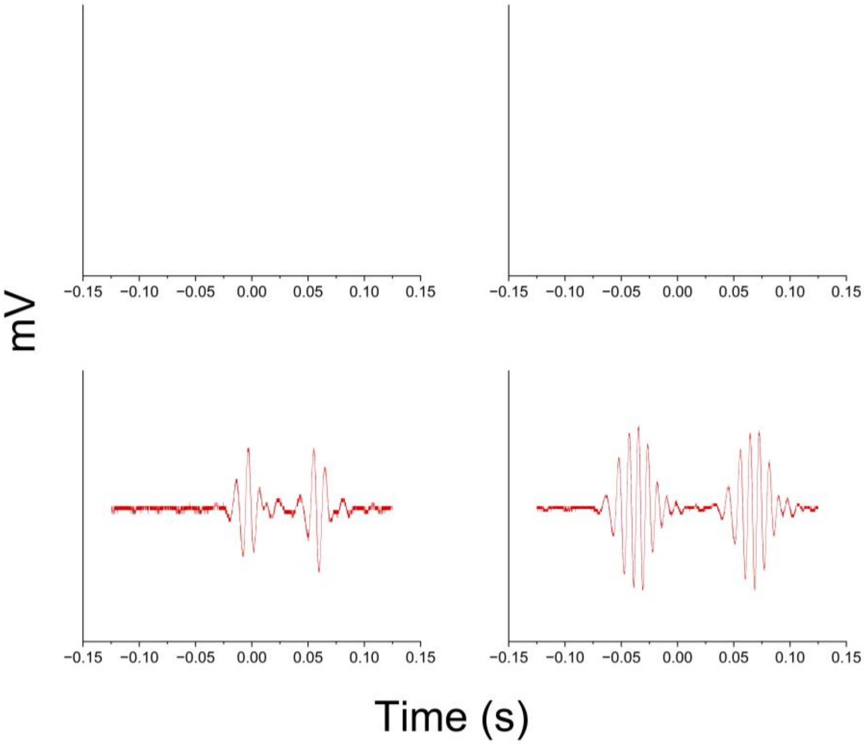

Figure 9S. Tactile sequential stimulations delivered by the Xiaomi MI A2 smartphone.

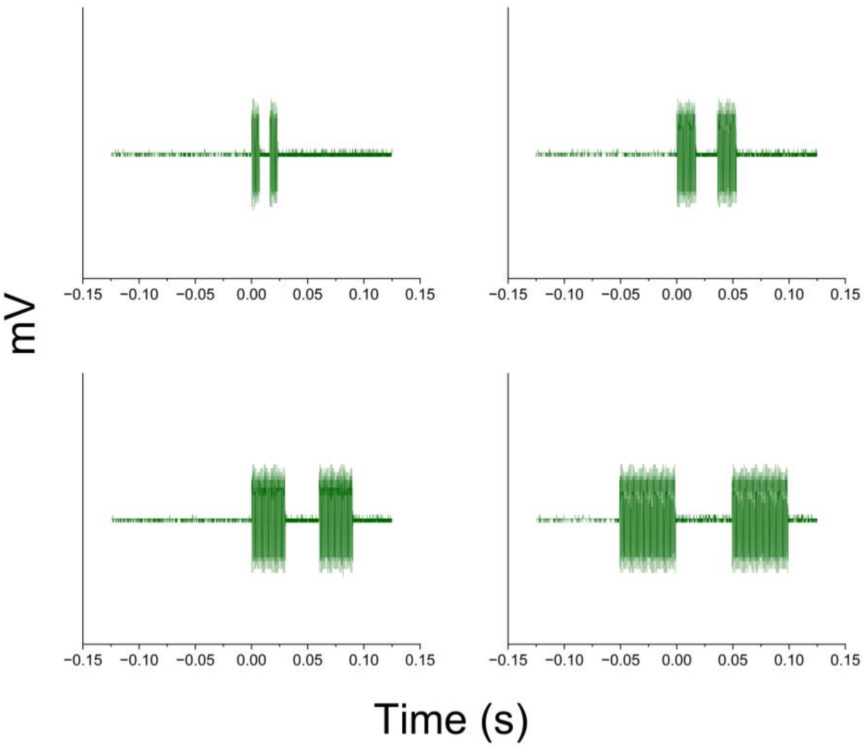

Figure 10S. Auditory sequential stimulations delivered by the Samsung A40 smartphone.

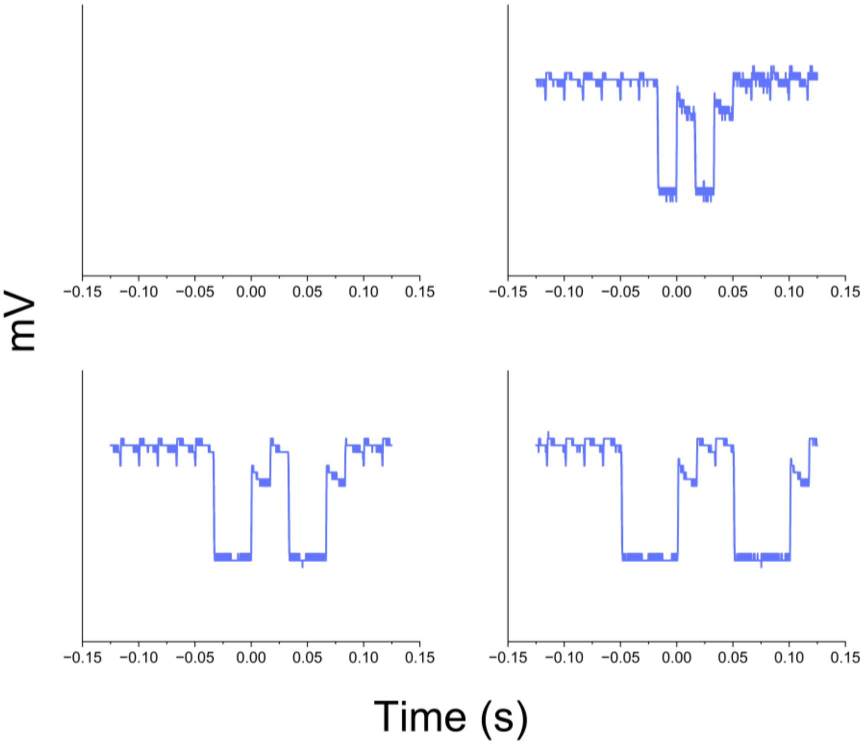

Figure 11S. Visual sequential stimulations delivered by the Samsung A40 smartphone.

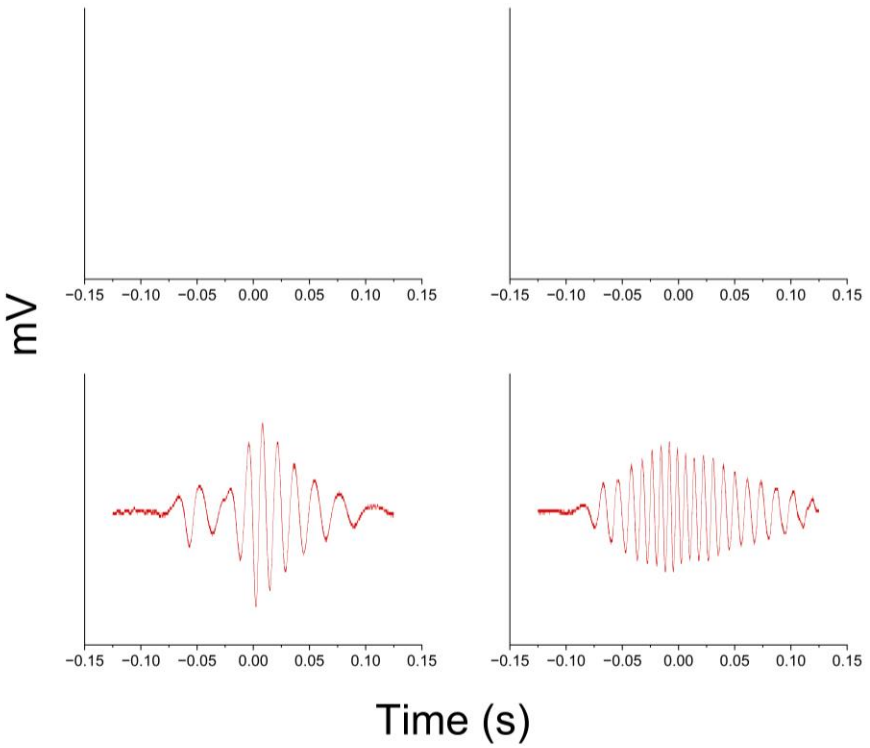

Figure 12S. Tactile sequential stimulations delivered by the Samsung A40 smartphone.

Bimodal Single Stimulations (referring to Figure 8 in the main text)

Expected durations are, from left to right according to row placement, 7, 17, 30, 50, and 100ms respectively.

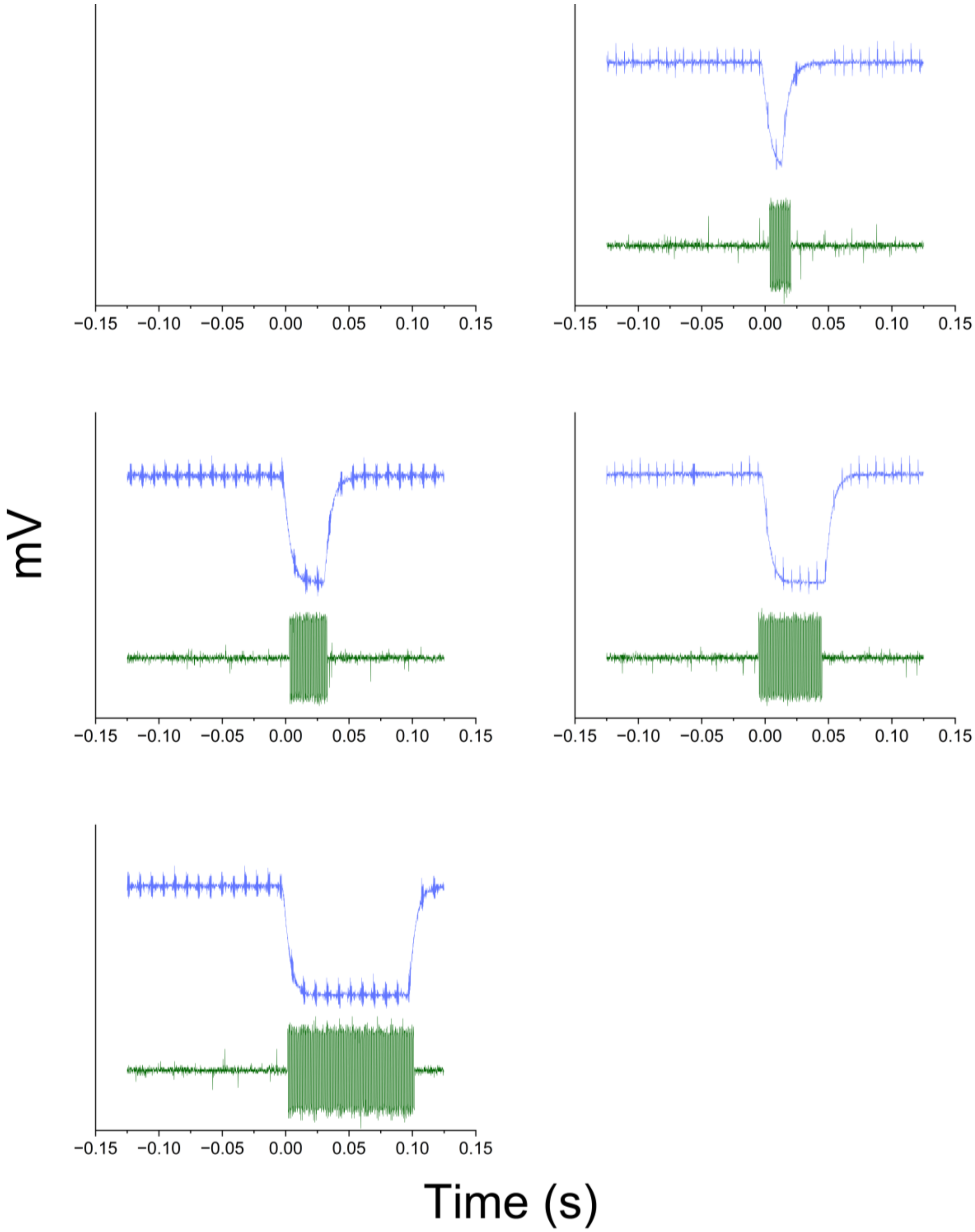

Figure 13S. Audiovisual single stimulations delivered by the Xiaomi MI A2 smartphone.

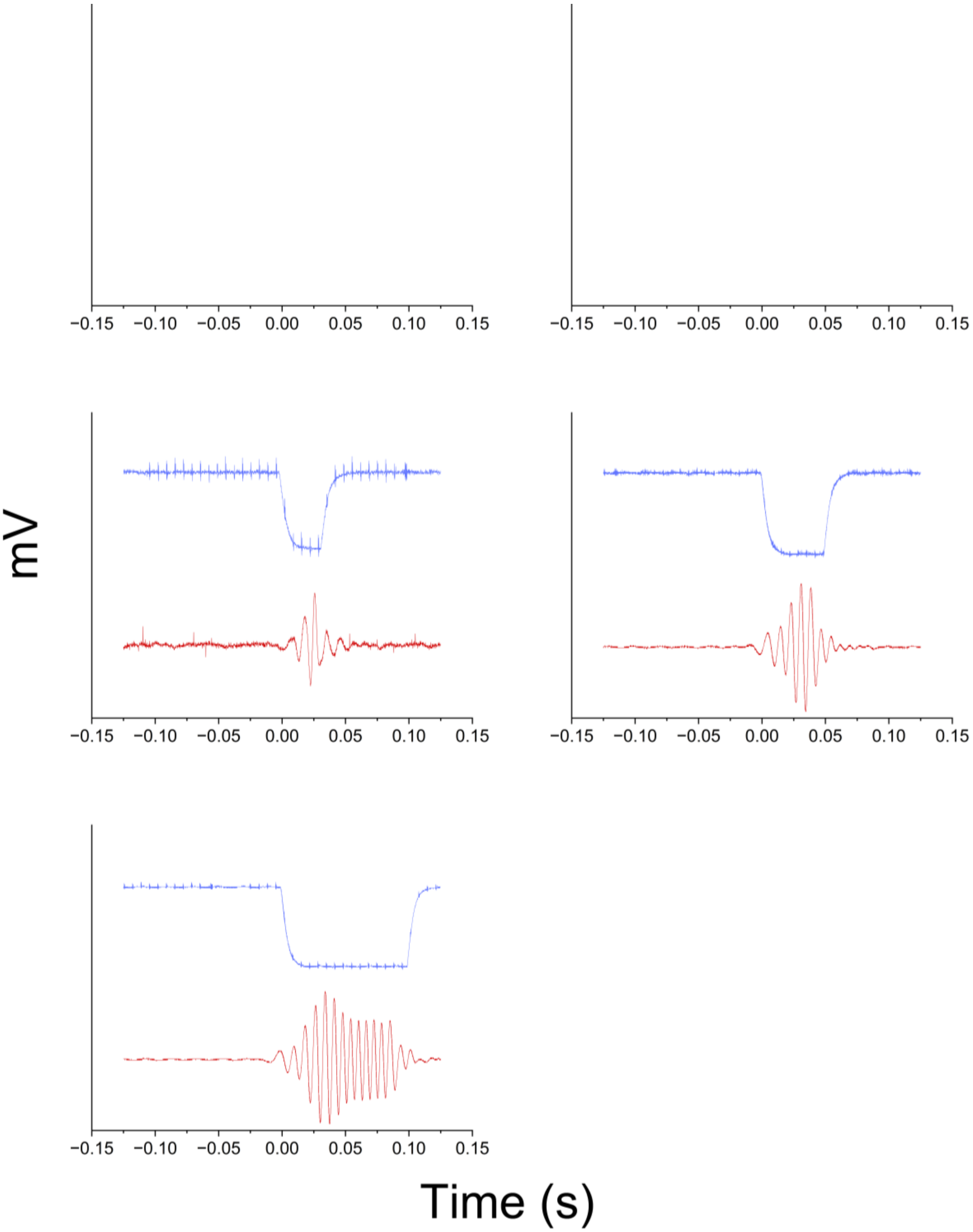

Figure 14S. Visuotactile single stimulations delivered by the Xiaomi MI A2 smartphone.

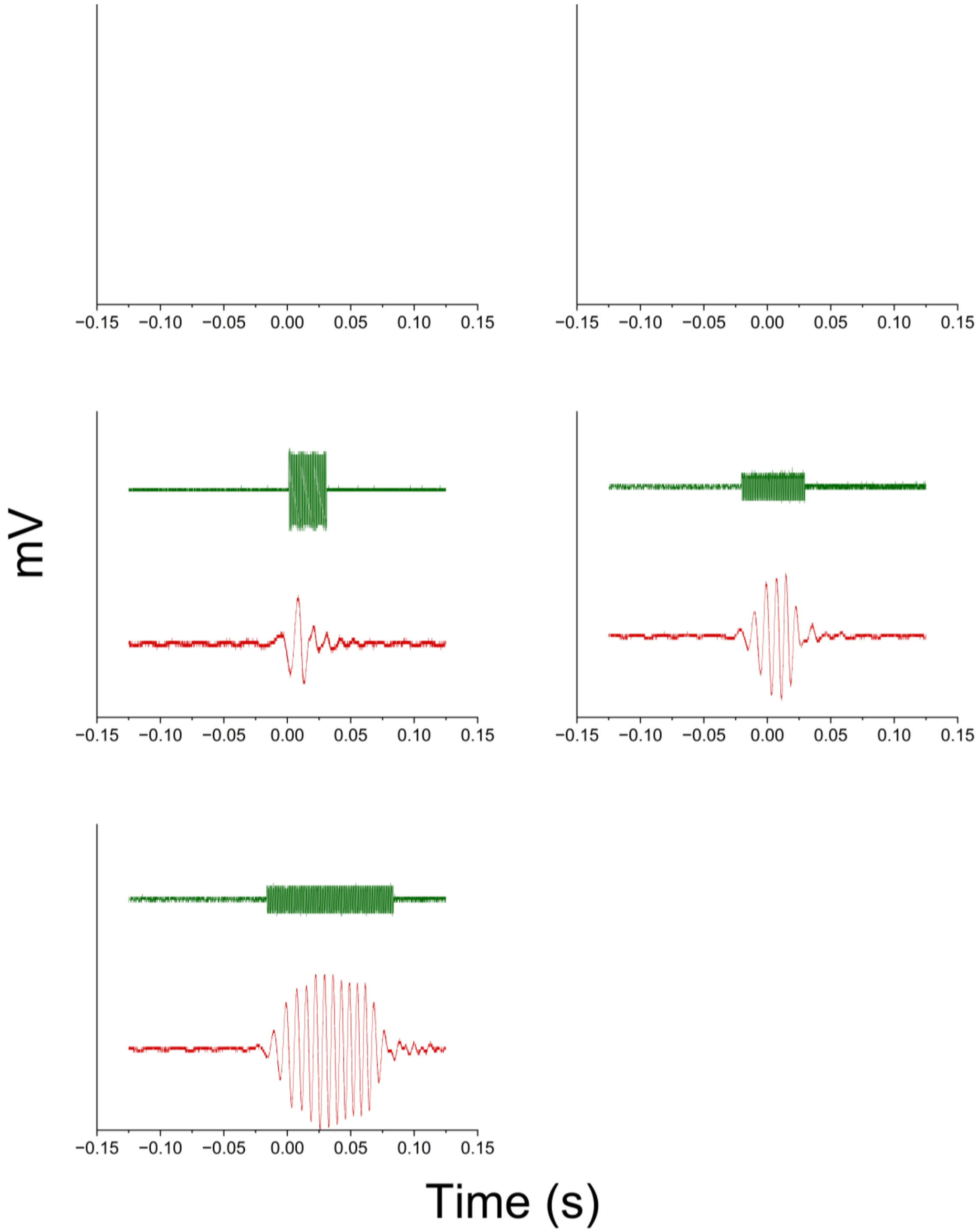

Figure 15S. Audiotactile single stimulations delivered by the Xiaomi MI A2 smartphone.

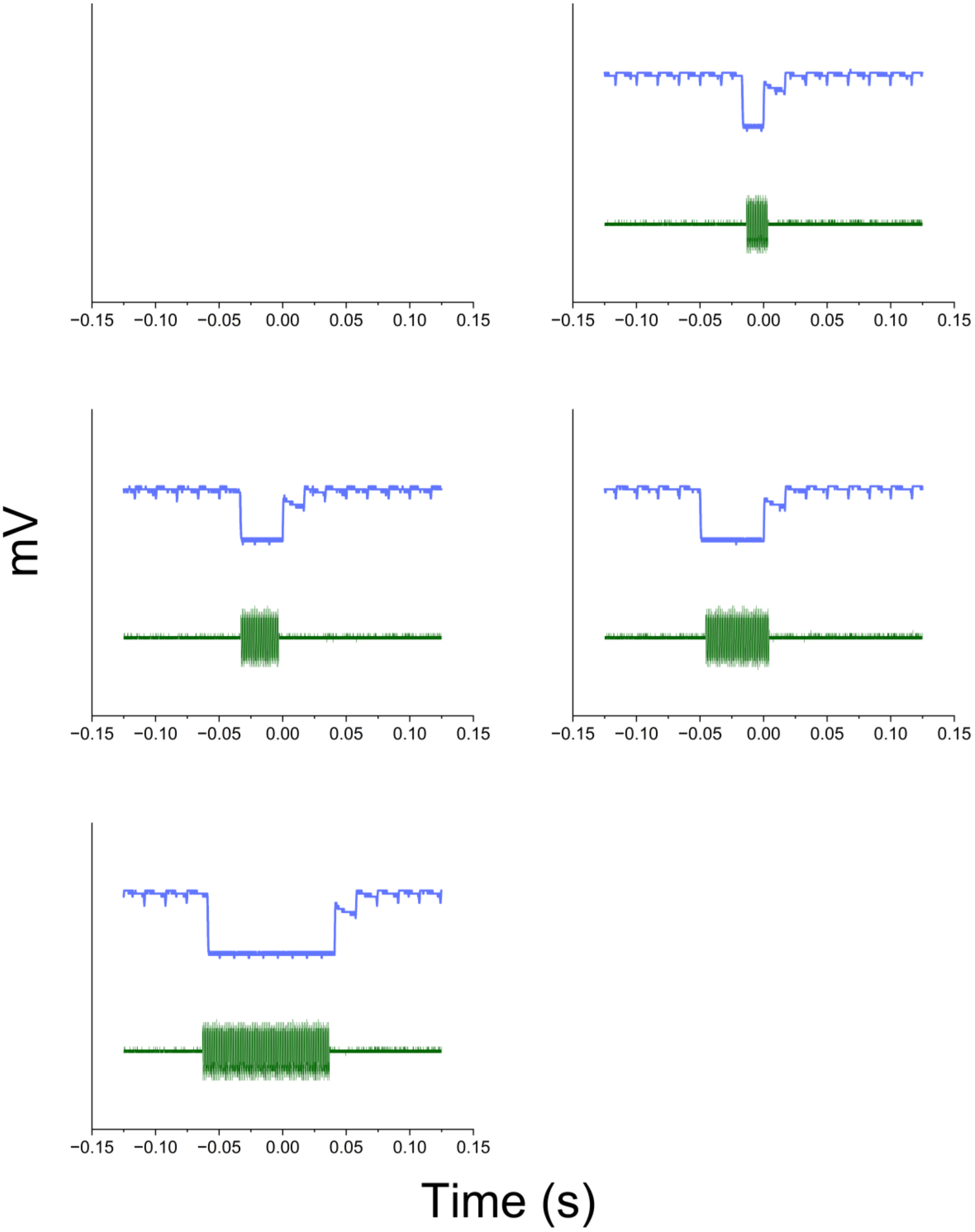

Figure 16S. Audiovisual single stimulations delivered by the Samsung A40 smartphone.

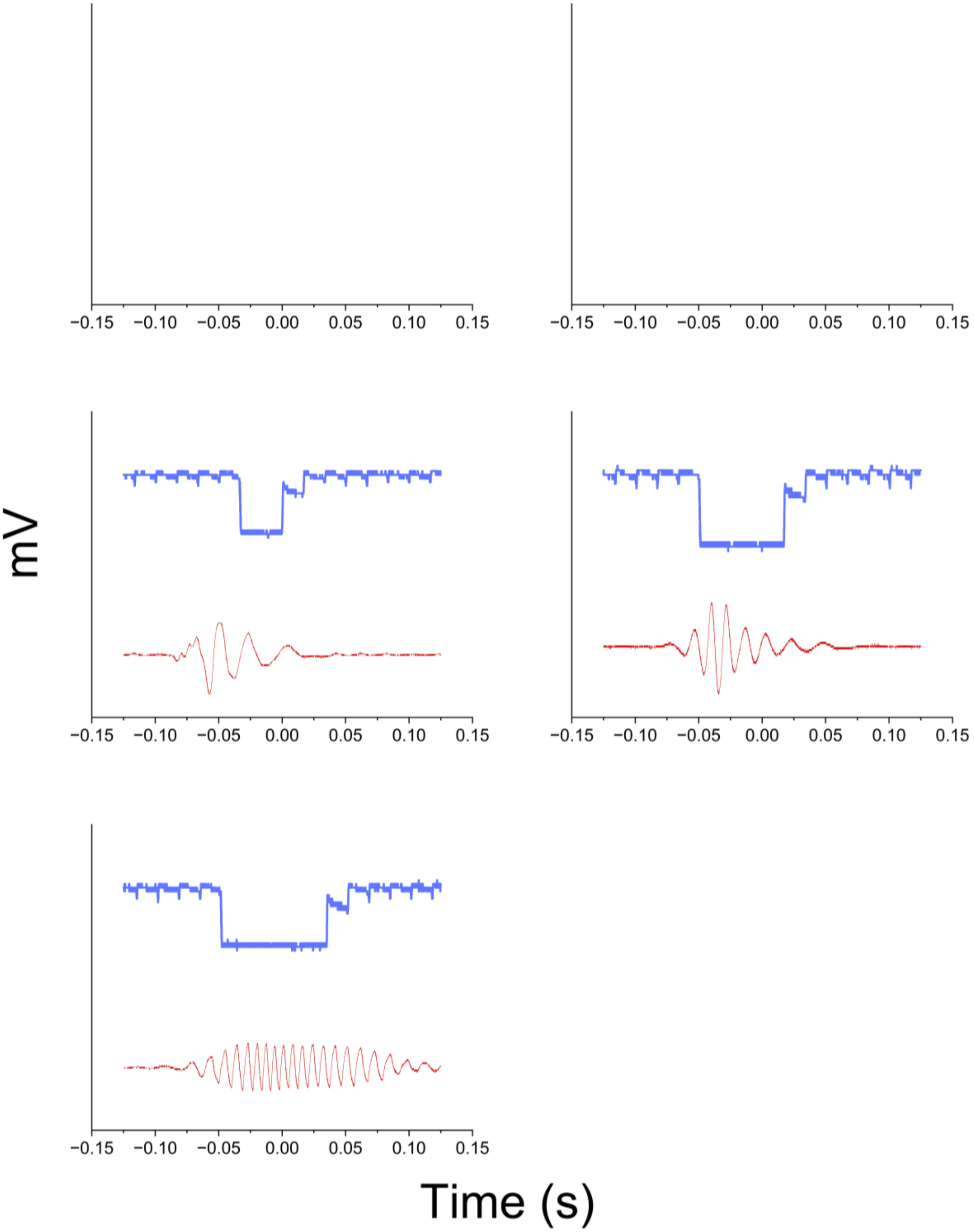

Figure 17S. Visuotactile single stimulations delivered by the Samsung A40 smartphone.

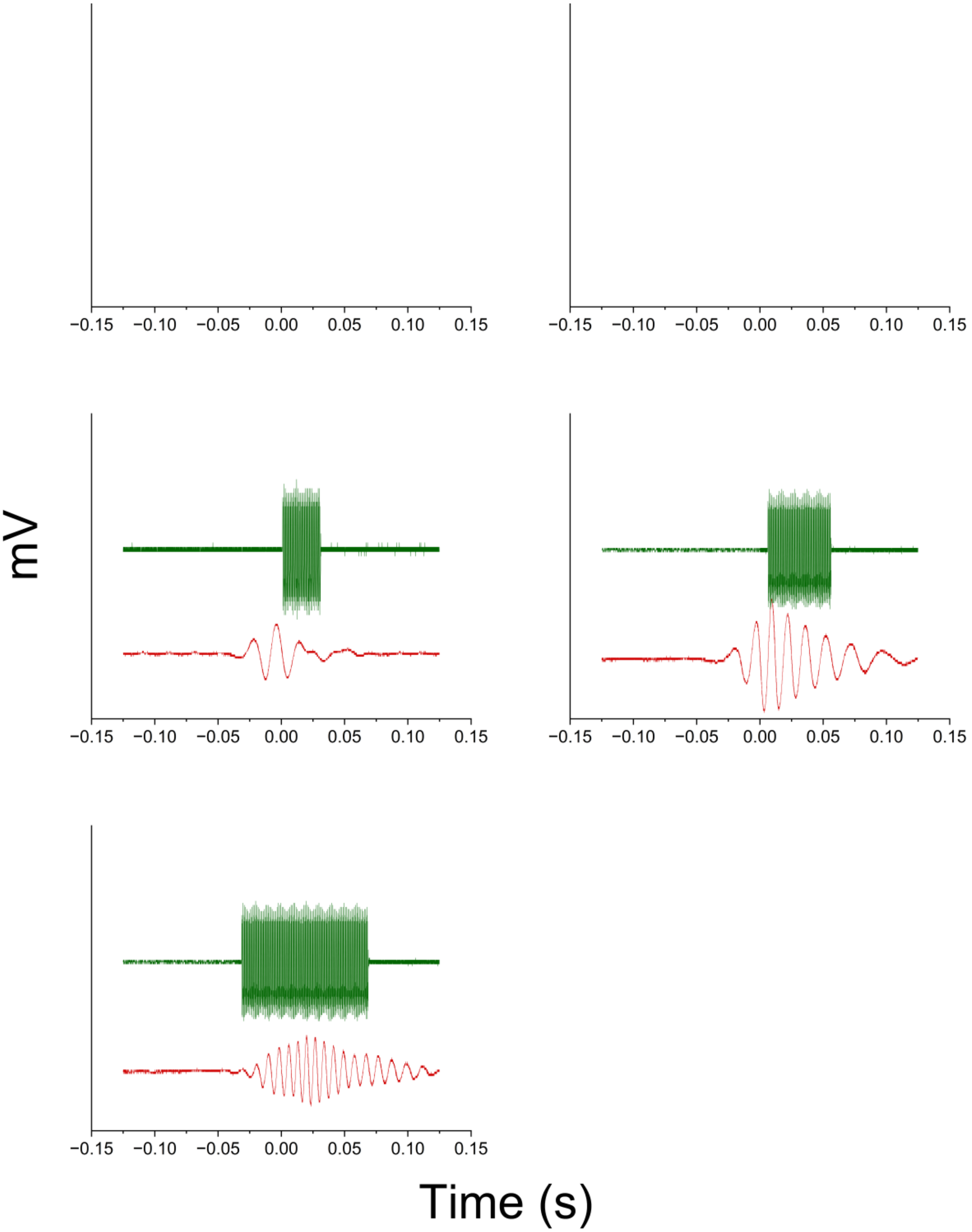

Figure 18S. Audiotactile single stimulations delivered by the Samsung A40 smartphone.

Bimodal Paired Stimulations (referring to Figure 9 in the main text)

Expected durations-interval pairs are, from left to right according to row placement, 7-14, 17-34, 30-60, and 50-100ms, respectively.

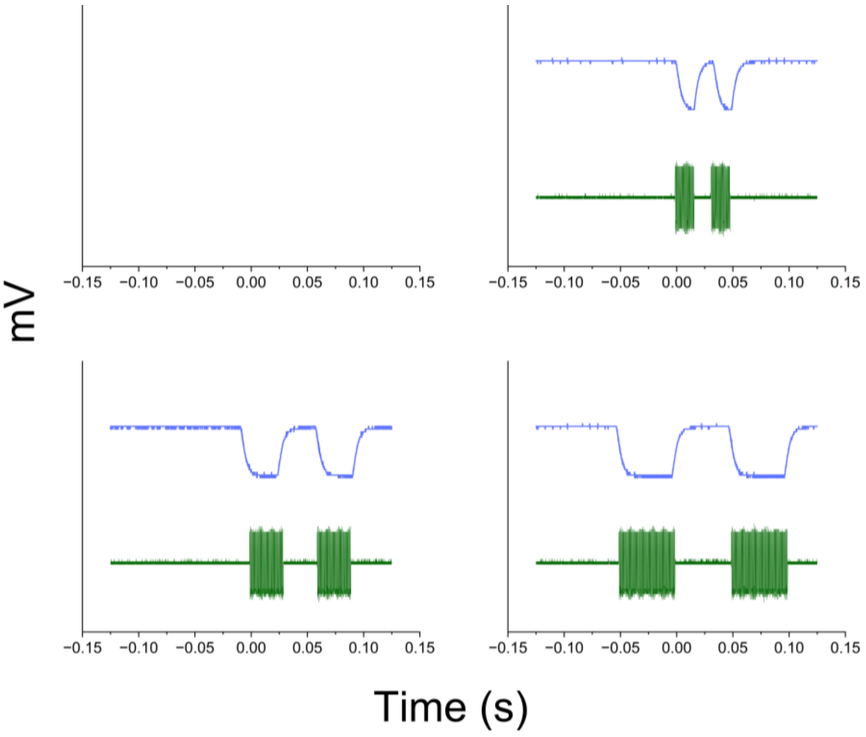

Figure 19S. Audiovisual sequential stimulations delivered by the Xiaomi MI A2 smartphone.

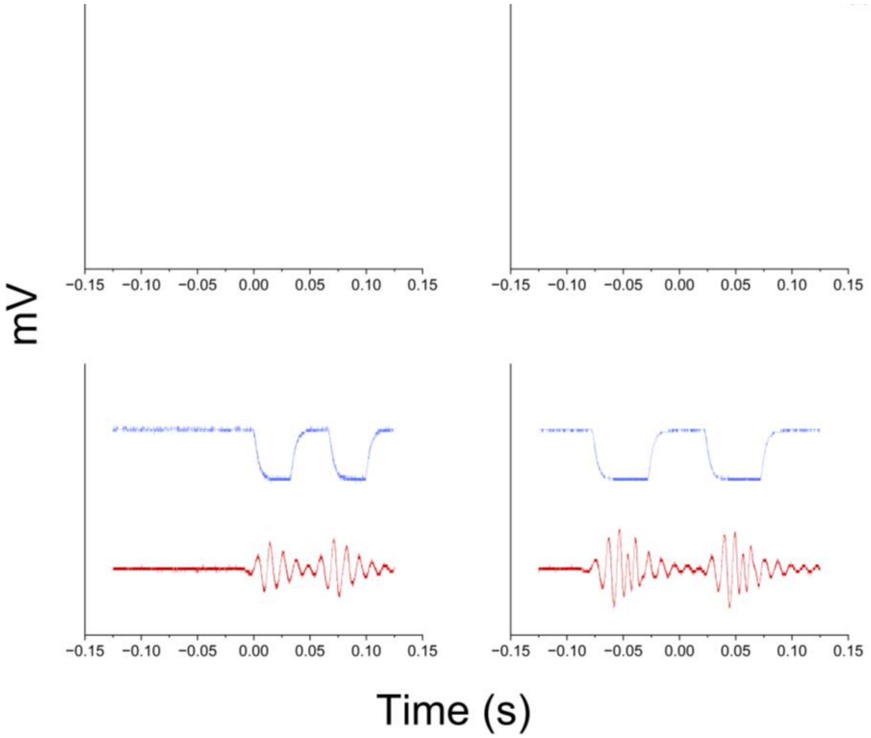

Figure 20S. Visuotactile sequential stimulations delivered by the Xiaomi MI A2 smartphone.

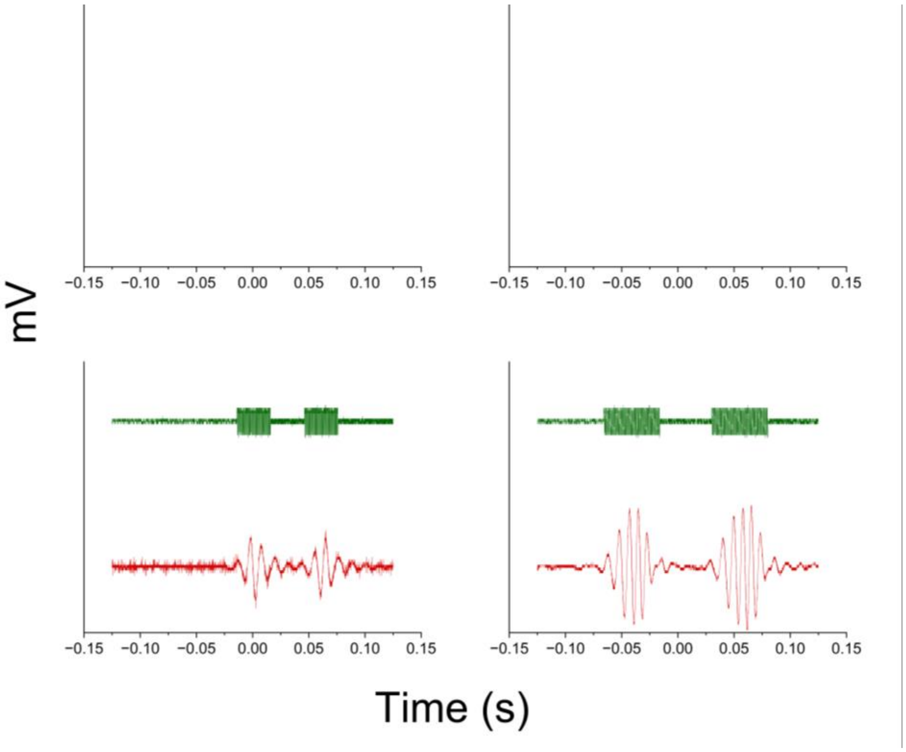

Figure 21S. Audiotactile sequential stimulations delivered by the Xiaomi MI A2 smartphone.

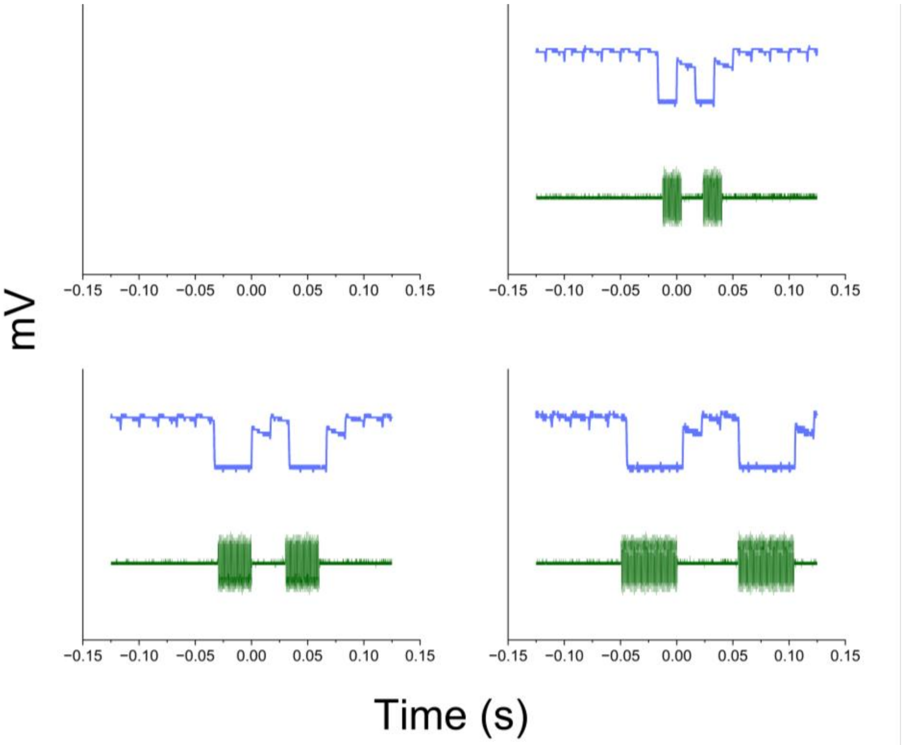

Figure 22S. Audiovisual sequential stimulations delivered by the Samsung A40 smartphone.

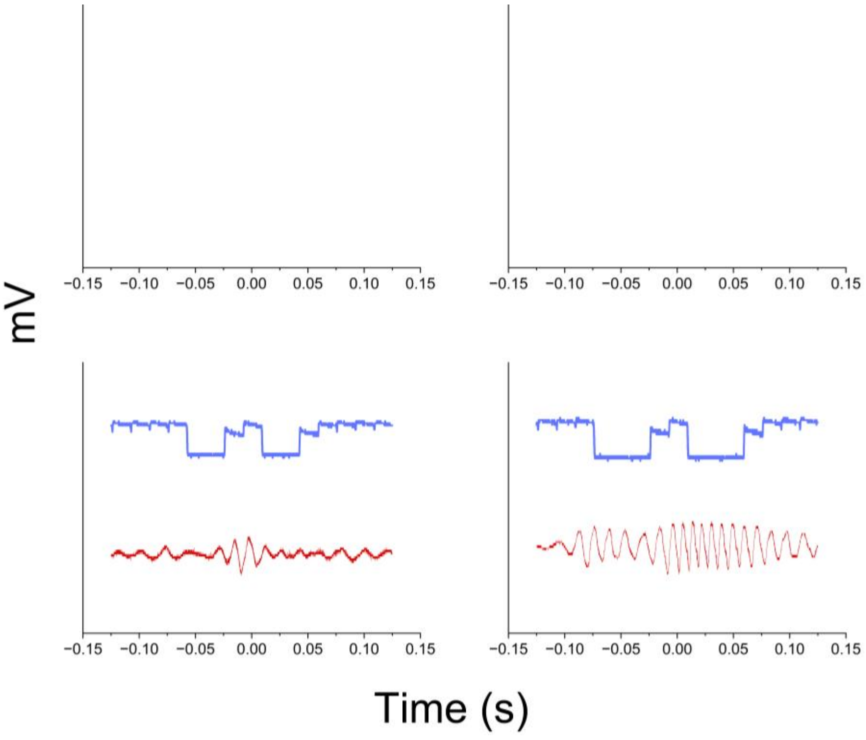

Figure 23S. Visuotactile sequential stimulations delivered by the Samsung A40 smartphone.

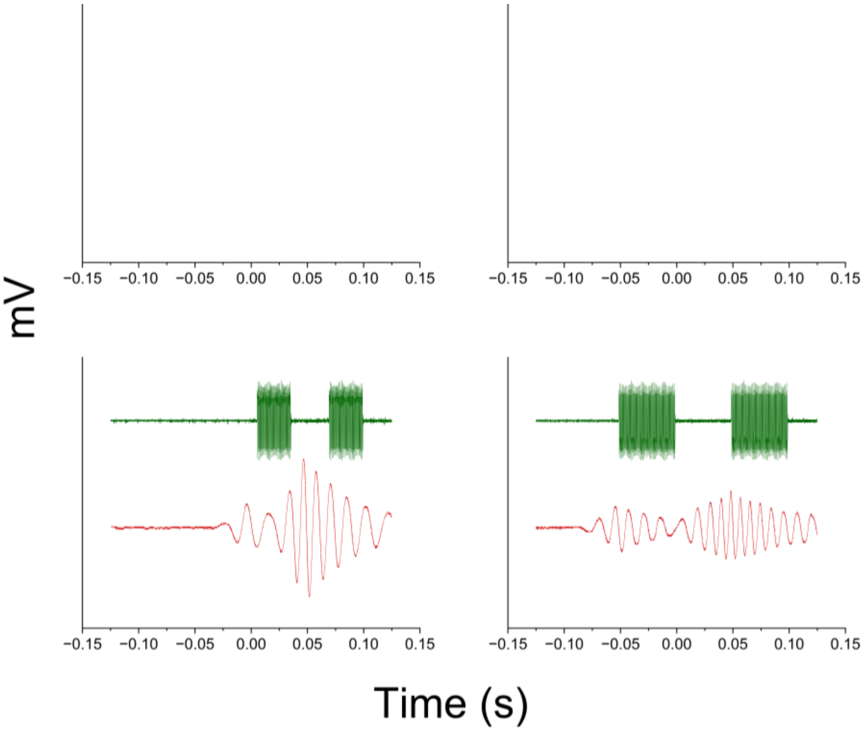

Figure 24S. Audiotactile sequential stimulations delivered by the Samsung A40 smartphone.

Trimodal Stimulations (referring to Figure 10 in the main text)

Expected durations are, from left to right according to row placement, 7, 17, 30, 50, and 100ms respectively.

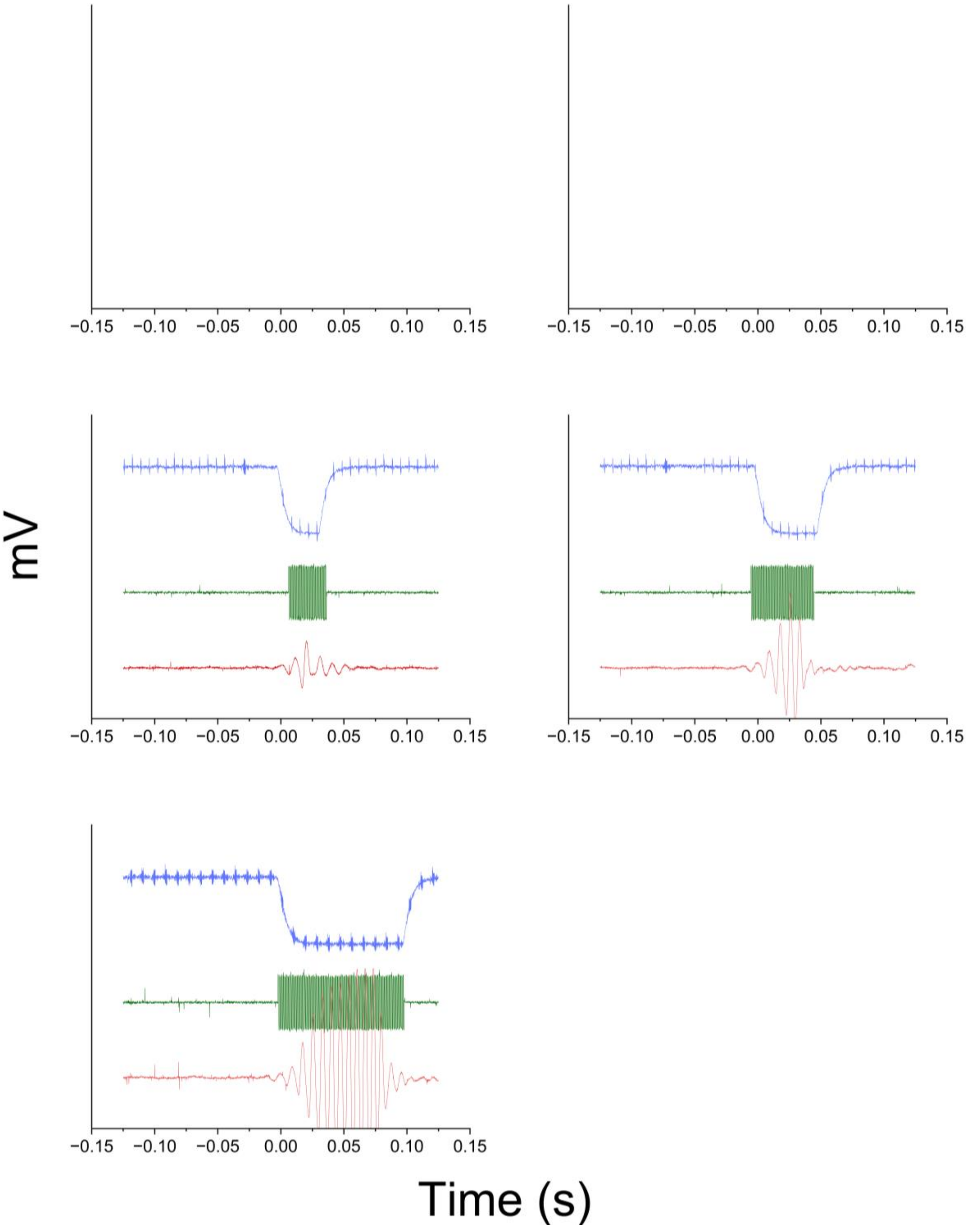

Figure 25S. Trimodal single stimulations delivered by the Xiaomi MI A2 smartphone.

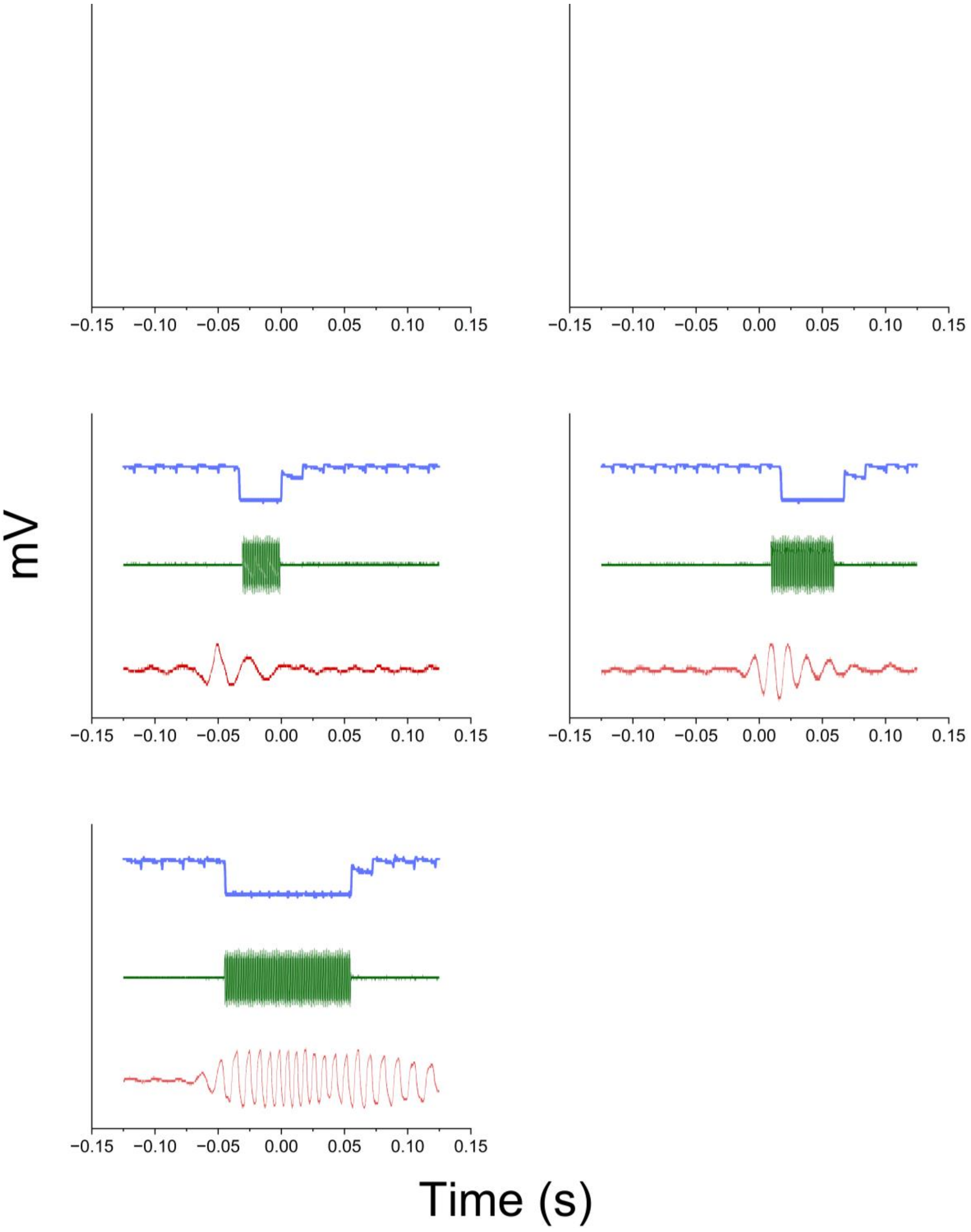

Figure 26S. Trimodal single stimulations delivered by the Xiaomi MI A2 smartphone.

Expected durations-interval pairs are, from left to right according to row placement, 7-14, 17-34, 30-60, and 50-100ms, respectively.

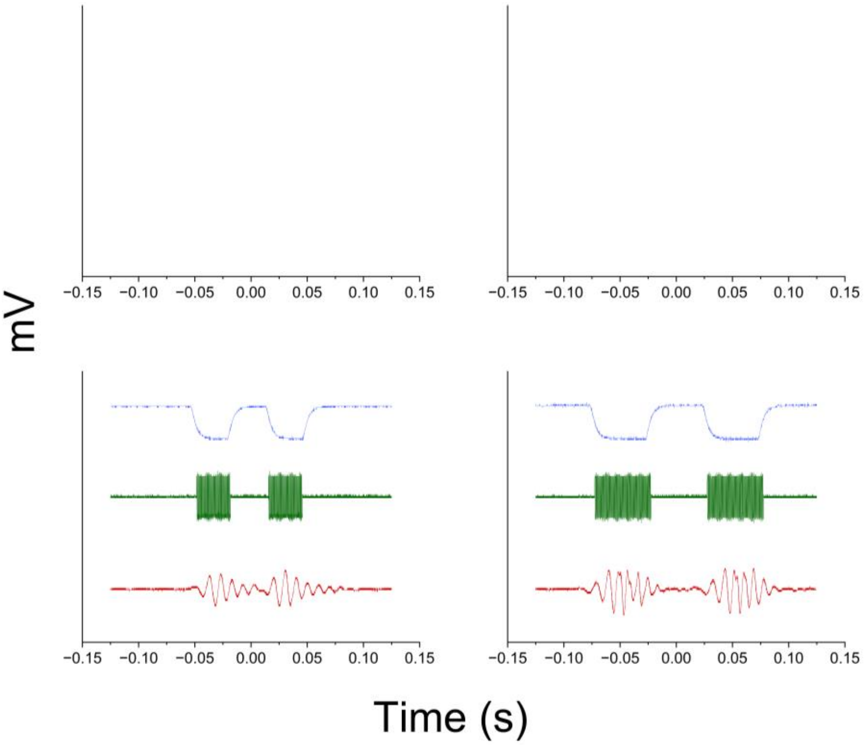

Figure 27S. Trimodal sequential stimulations delivered by the Xiaomi MI A2 smartphone.

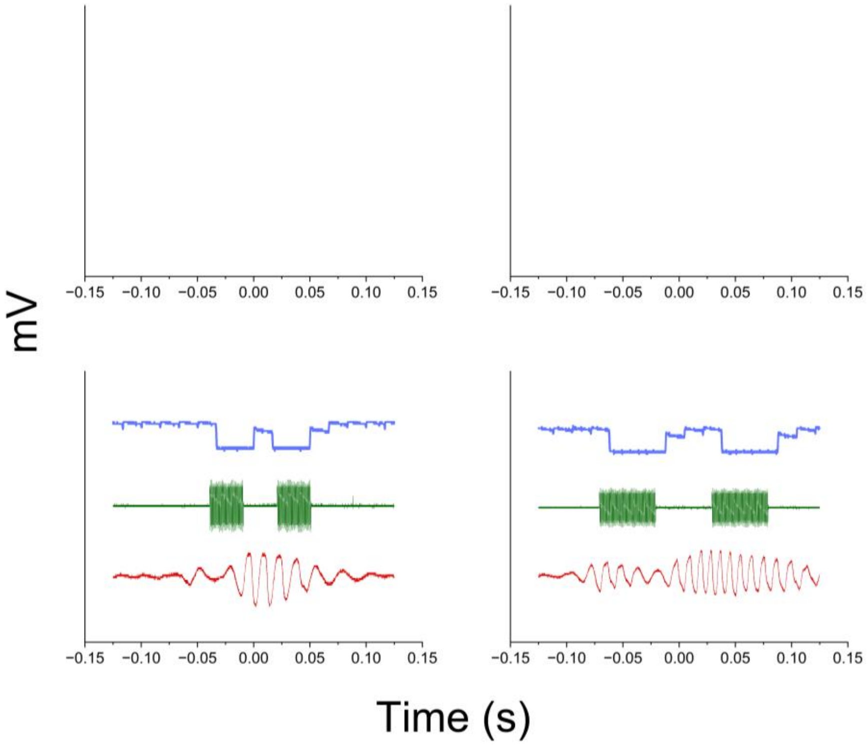

Figure 28S. Trimodal sequential stimulations delivered by the Samsung A40 smartphone.
